# Supplementary material for: OTULIN Antagonizes LUBAC Signaling by Specifically Hydrolyzing Met1-Linked Polyubiquitin
Source: Cell. 2013 Jun 6;153(6):1312–26. doi: 10.1016/j.cell.2013.05.014 (PMC3690481; doi:10.1016/j.cell.2013.05.014)
Supplement: Table S1. Data Collection and Refinement Statistics, Related to Figure 2 [file mmc1.pdf]

Table S1. Data Collection and Refinement Statistics

	OTULIN 80-352 SeMet	OTULIN 80-352	OTULIN 80-352 D336A	OTULIN 80-352 C129A •Met1-diUb
**Data collection**				
Beamline	ESRF ID23-1	ESRF ID23-1	ESRF ID14-4	Diamond I04
Space group	$P2_12_12_1$	$P2_12_12_1$	$P2_12_12_1$	$H3_2$
a, *b*, *c* (Å)	43.59, 72.44, 95.19	43.49, 72.01, 94.67	43.81, 72.20, 94.84	100.02, 100.02, 280.26
α, β, γ (°)	90, 90, 90	90, 90, 90	90, 90, 90	90, 90, 120
Wavelength	0.9794 (Peak)	0.9794	0.9762	0.9795
Resolution (Å)	57.65-2.9 (3.06-2.90)	39.5-1.30 (1.32-1.30)	47.42-1.35 (1.37-1.35)	42.80-1.90 (2.00-1.90)
R_{merge}	0.151 (0.311)	0.113 (0.326)	0.094 (0.705)	0.094 (0.778)
$I / \sigma I$	14.8 (8.1)	7.3 (3.3)	9.9 (2.4)	14.4 (3.0)
Completeness (%)	100 (100)	99.8 (99.4)	100 (100)	100 (100)
Redundancy	13.7 (13.5)	3.9 (3.9)	6.1 (4.7)	8.9 (9.1)
**Phasing**				
Resolution (Å)	57.65-2.90 (3.06-2.90)			
Anomalous completeness	100 (100)			
Anomalous multiplicity	7.5 (7.2)			
<FOM>	0.18			
<FOM>$_{DM}$extended to 2.23 Å	0.91			
**Refinement**				
Resolution (Å)		33.58-1.30	47.42-1.35	42.80-1.90
No. reflections		69951	66797	42965
R_{work} / R_{free}		12.6/15.3	12.3/15.8	18.4/22.0
No. atoms				
Protein		2345	2337	3349
Ligand/ion		20	20	35
Water		301	319	371
B-factors				
Wilson *B*		16.2	18.0	27.4
Protein		15.4	18.2	31.4
Ligand/ion		25.2	33.5	55.9
Water		27.1	31.5	39.1
R.m.s deviations				
Bond lengths (Å)		0.023	0.022	0.007
Bond angles (°)		1.712	1.785	1.020
Ramachandran statistics (favoured / allowed / outliers)		97.6/ 2.4 / 0	97 / 3 / 0	97.6 / 2.4 / 0

Numbers in brackets are for the highest resolution bin.
