# Supplementary material for: OTULIN Antagonizes LUBAC Signaling by Specifically Hydrolyzing Met1-Linked Polyubiquitin
Source: Cell. 2013 Jun 6;153(6):1312–26. doi: 10.1016/j.cell.2013.05.014 (PMC3690481; doi:10.1016/j.cell.2013.05.014)
Supplement: Document S1. Article plus Supplemental Information [file mmc2.pdf]

Cell

OTULIN Antagonizes LUBAC Signaling by Specifically Hydrolyzing Met1-Linked Polyubiquitin

Kirstin Keusekotten,[1,5] Paul Ronald Elliott,[1,5] Laura Glockner,[2] Berthe Katrine Fiil,[3] Rune Busk Damgaard,[3] Yogesh Kulathu,[1] Tobias Wauer,[1] Manuela Kathrin Hospenthal,[1] Mads Gyrd-Hansen,[3] Daniel Krappmann,[2] Kay Hofmann,[4] and David Komander[1,*]
[1]Medical Research Council Laboratory of Molecular Biology, Francis Crick Avenue, Cambridge, CB2 0QH, UK
[2]Helmholtz Zentrum München, German Research Center for Environmental Health, Research Unit Cellular Signal Integration, Institute of Molecular Toxicology and Pharmacology, Ingolstädter Landstrasse 1, 85764 Neuherberg, Germany
[3]Department of Disease Biology, Novo Nordisk Foundation Center for Protein Research, University of Copenhagen, 2200 Copenhagen, Denmark
[4]Institute for Genetics, University of Cologne, Zülpicher Strasse 47a, 50674 Cologne, Germany
[5]These authors contributed equally to this work
*Correspondence: dk@mrc-lmb.cam.ac.uk
http://dx.doi.org/10.1016/j.cell.2013.05.014

SUMMARY

The linear ubiquitin (Ub) chain assembly complex (LUBAC) is an E3 ligase that specifically assembles Met1-linked (also known as linear) Ub chains that regulate nuclear factor κB (NF-κB) signaling. Deubiquitinases (DUBs) are key regulators of Ub signaling, but a dedicated DUB for Met1 linkages has not been identified. Here, we reveal a previously unannotated human DUB, OTULIN (also known as FAM105B), which is exquisitely specific for Met1 linkages. Crystal structures of the OTULIN catalytic domain in complex with diubiquitin reveal Met1-specific Ub-binding sites and a mechanism of substrate-assisted catalysis in which the proximal Ub activates the catalytic triad of the protease. Mutation of Ub Glu16 inhibits OTULIN activity by reducing k_{cat} 240-fold. OTULIN overexpression or knockdown affects NF-κB responses to LUBAC, TNFα, and poly(I:C) and sensitizes cells to TNFα-induced cell death. We show that OTULIN binds LUBAC and that overexpression of OTULIN prevents TNFα-induced NEMO association with ubiquitinated RIPK1. Our data suggest that OTULIN regulates Met1-polyUb signaling.

INTRODUCTION

Ubiquitination is an important posttranslational modification that regulates diverse processes, including protein degradation, intracellular trafficking, transcription, kinase activation, and the DNA damage response (Hershko and Ciechanover, 1998; Komander and Rape, 2012). This variety of functions is mediated by eight different types of polyubiquitin (polyUb) linkages, and, although the roles of Lys48- and Lys63-linked polyUb have been studied in great detail, much less is known about the remaining "atypical" Ub chains (Behrends and Harper, 2011; Kulathu and Komander, 2012).

Met1-linked polyUb (Met1-polyUb) is the source of the cellular Ub pool, given that Ub is translated as a polyprotein (Ozkaynak et al., 1984) and posttranslationally processed by dedicated DUBs, such as USP5 (also known as IsoT) (Amerik AYu et al., 1997). This chain type can also be assembled by the linear Ub chain assembly complex (LUBAC), a multisubunit E3 ligase consisting of HOIP, HOIL-1L, and SHARPIN (Gerlach et al., 2011; Ikeda et al., 2011; Kirisako et al., 2006; Tokunaga et al., 2011). LUBAC has roles in NF-κB activation (Haas et al., 2009; Tokunaga and Iwai, 2012; Tokunaga et al., 2009; Walczak et al., 2012) and is required for full activation of the inhibitor of κB (IκB) kinase (IKK) complex. IKK activation leads to the phosphorylation and degradation of IκB and the activation of the NF-κB transcription factor (Karin and Ben-Neriah, 2000). It is not fully understood how Met1-polyUb regulates this process, but it involves the binding and modification of the IKK subunit NEMO with Met1-linked chains. NEMO harbors a Met1-specific Ub-binding domain (UBD) that is important for NF-κB signaling (Komander et al., 2009; Rahighi et al., 2009).

Much less is known about DUBs that regulate Met1-polyUb chains, and a specific DUB for Met1-linkages has not been identified. Of the roughly 80 active DUBs in the human genome, many show weak or no activity toward Met1-linked chains (Faesen et al., 2011; Komander et al., 2009). A potential reason is the distinct chemistry of a peptide versus an isopeptide linkage (Figure 1A).

Ovarian tumor (OTU) domain DUBs regulate important cell-signaling pathways. A20 regulates NF-κB signaling (Hymowitz and Wertz, 2010), OTUD5 (also known as DUBA) regulates IRF3 signaling (Kayagaki et al., 2007), and OTUB1 regulates the DNA damage response (Nakada et al., 2010). OTU DUBs can be linkage specific. Structural work has revealed the basis for OTUB1 Lys48 specificity (Juang et al., 2012; Wiener et al.,

 ©2013 Elsevier Inc.

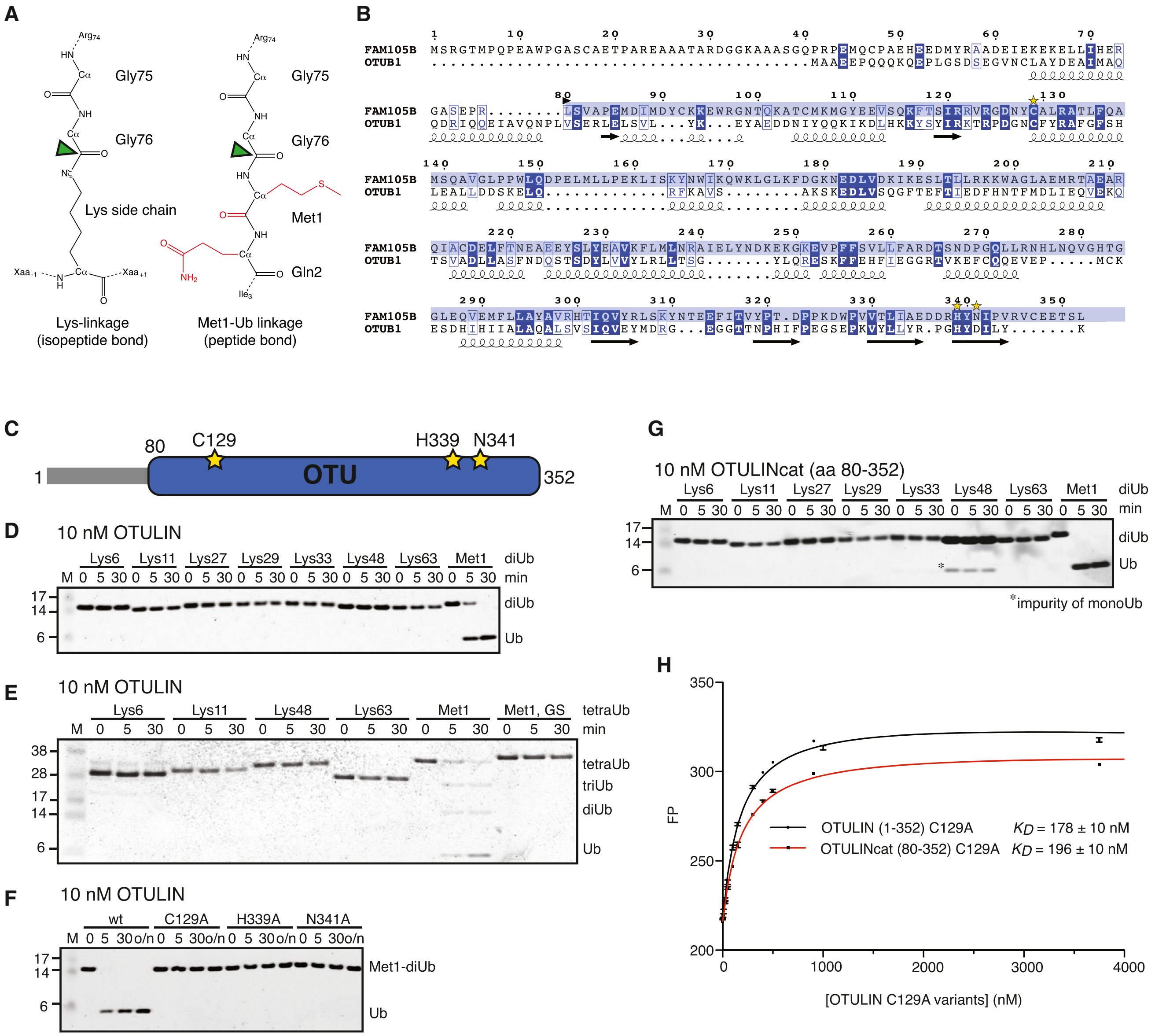

Figure 1. Identification and Specificity of OTULIN

(A) Chemical difference between an isopeptide (left) and Met1-peptide linkage (right) in diUb. The distal Ub (top) is linked via its C-terminal Gly^{75}-Gly^{76} sequence to a Lys side chain ε-amino group in another Ub or on a substrate, generating a branched peptide. In a Met1-linked chain, the C-terminal Gly76 is connected to Met1 of the distal Ub in a standard peptide linkage. The Met1 and Gln2 side chains, as well as the Met1 carbonyl (red), represent steric differences in comparison to an isopeptide linkage. A green arrow indicates the scissile bond in a DUB reaction.

(B) Sequence alignment of FAM105B/OTULIN with OTUB1. Sequence identity is 18% for the catalytic domain. Secondary structure elements are shown for OTUB1. The OTU domain is indicated in blue, and catalytic residues are labeled with yellow stars.

(C) The domain structure of OTULIN colored as in (B).

(D) Linkage specificity of OTULIN. diUb (1 μM) of all possible linkage types is hydrolyzed over a time course by 10 nM OTULIN and visualized on silver-stained 4%–12% gradient SDS-PAGE gels. See Figure S1D for the assay at 1 μM OTULIN concentration.

(E) Cleavage of tetraUb chains, as in (D). The last substrate is a Met1-tetraUb with G76S mutation in all Ub moieties.

(F) Hydrolysis of Met1-diUb by OTULIN wild-type (WT) and catalytic mutants as indicated.

(G) The OTU domain of OTULIN encodes Met1-linked Ub specificity. diUb specificity analysis as in (D) with OTULIN 80–352 at a 10 nM concentration.

(H) Affinity measurements by fluorescence anisotropy with OTULIN (1–352) C129A or OTULIN (80–352) C129A and FlAsH-tagged Met1-diUb, as described in the Extended Experimental Procedures. Error bars represent SD from the mean of measurements performed in triplicate.

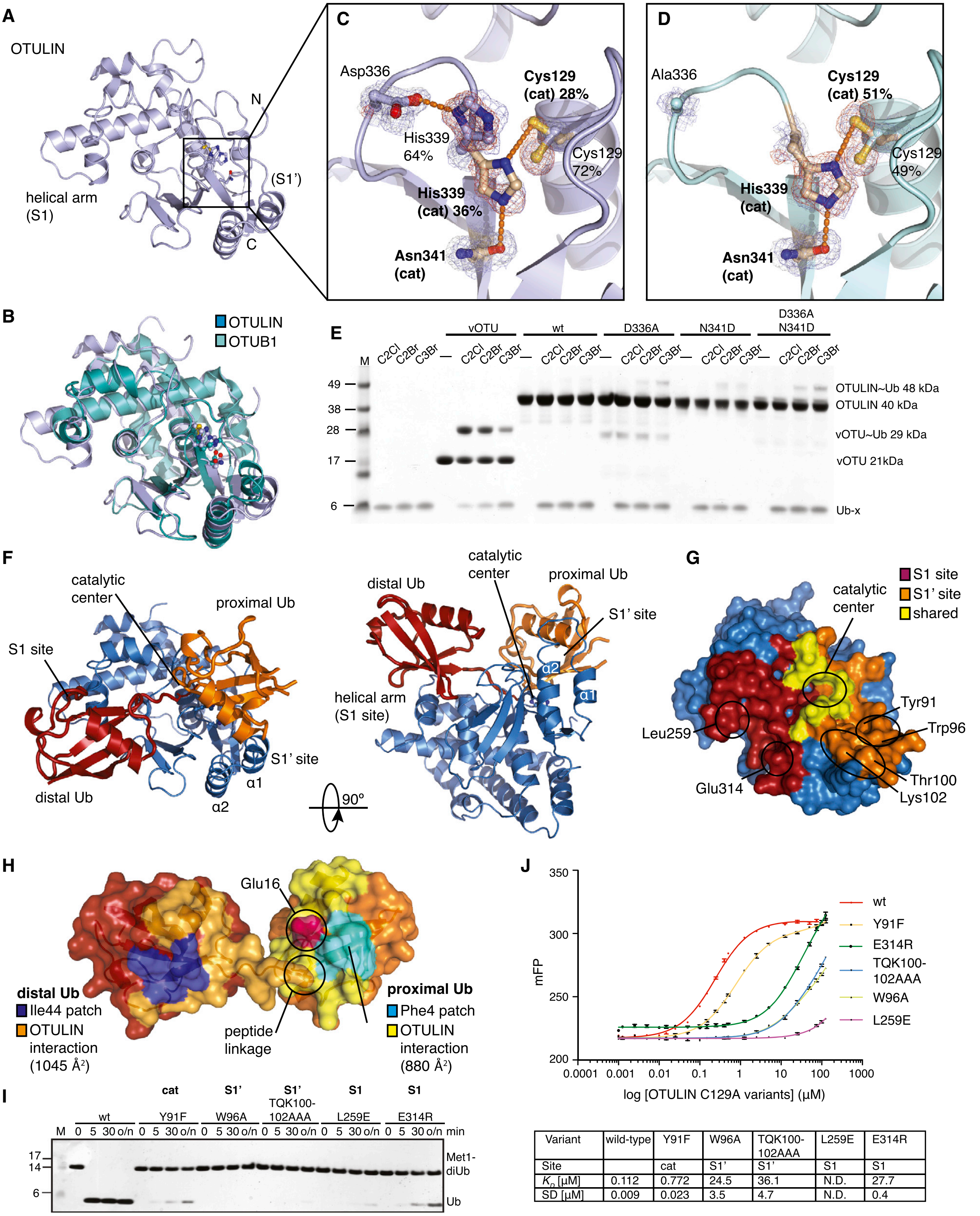

Variant	wild-type	Y91F	W96A	TQK100-102AAA	L259E	E314R
Site		cat	S1'	S1'	S1	S1
K_D [μM]	0.112	0.772	24.5	36.1	N.D.	27.7
SD [μM]	0.009	0.023	3.5	4.7	N.D.	0.4

(legend on next page)

2012) and TRABID specificity against Lys29 and Lys33 linkages (Licchesi et al., 2012). Moreover, viral OTU DUBs have been reported that are highly divergent in sequence but are structurally similar (Frias-Staheli et al., 2007).

Here, we identify a previously unannotated human DUB, FAM105B/OTULIN, which is specific for Met1-linked Ub chains. Structural studies reveal that this specificity is due to Met1-specific Ub-binding sites and a mechanism of substrate-assisted catalysis where a residue in a Met1-linked chain directly participates in the organization of the catalytic triad of the enzyme. Overexpression and knockdown analysis of OTULIN suggest that the protein binds LUBAC and regulates LUBAC-mediated processes in cells.

RESULTS

FAM105B/OTULIN, a Met1-Linkage-Specific OTU DUB

Given the high sequence divergence of OTU domains, we set out to identify unstudied OTU enzymes using a bioinformatical screen based on generalized profile analysis (Bucher et al., 1996). Iterative profile refinement, starting from a multiple-sequence alignment of experimentally validated OTUs, indicated an OTU domain with a complete catalytic triad in the uncharacterized human protein FAM105B (Figure 1B). FAM105B comprises 352 amino acids (aa), and the OTU domain spans the majority of the protein (aa 80–352) and an N-terminal region with predicted helical content (Figure 1B, 1C). The catalytic domain is highly conserved between species (Figure S1A available online). Bacterially expressed full-length FAM105B did not hydrolyze common fluorescent substrates such as Ub-AMC (Figure S1B). Ub-based suicide inhibitors that comprise an electrophilic group at the Ub C terminus (Borodovsky et al., 2002) covalently modify most OTU domain DUBs but showed no reactivity against FAM105B (Figure S1C). However, DUB assays against diubiquitin (diUb) of all eight linkage types revealed that FAM105B exclusively hydrolyzed Met1-diUb (Figure 1D). The enzyme was active at 10 nM concentration (Figure 1D) and remained Met1 linkage specific at a 1 μM concentration (Figure S1D). Specificity is maintained when longer Ub chains are used as substrates (Figure 1E) but depended on an intact Ub Gly^{76}-Met^{1} linkage sequence between Ub moieties, given that mutant tetraUb with Ser^{76}-Met^{1} linkages was not hydrolyzed (Figures 1E and S1E). Catalytic mutants of FAM105B (C129A, H339A, and N341A) did not hydrolyze Met1-diUb (Figure 1F). Having established FAM105B as a Met1-linkage-specific OTU DUB, we named the enzyme OTULIN (OTU DUB with linear linkage specificity). OTULIN is unique, given that the 14 annotated human OTU DUBs cannot hydrolyze Met1-diUb (Mevissen et al., 2013).

Molecular Basis for OTULIN Specificity

Structural studies revealed how OTULIN achieved its unique specificity for Met1 linkages. The catalytic domain of OTULIN (OTULINcat, aa 80–352) is sufficient for linkage specificity (Figure 1G), and OTULINcat C129A bound Met1-diUb with a similarly high affinity as full-length OTULIN C129A, as revealed by fluorescence anisotropy measurements (K_D 196 versus 178 nM, Figure 1H).

OTULINcat was crystallized, and its structure was determined to 1.3 Å resolution with SeMet phasing (Figure 2A and Table S1). OTULINcat adopts an OTU fold most similar to OTUB1 (root-mean-square deviation [rmsd] 2.1 Å, DALI *Z* score 8.7) (Figure 2B). Interestingly, catalytic triad residues His339 and Cys129 display two alternate conformations. In the "active" conformation (occupancy ~30%), the catalytic triad is formed by interactions between Asn341, His339, and Cys129 (Figure 2C); e.g., as observed in OTUB1 in complex with Ub suicide inhibitor (Wiener et al., 2012) (Figures S2A–S2C). In the "inhibited" conformation (occupancy ~70%), Asp336 pulls His339 away from its catalytic position (Figure 2C), and Cys129 flips to an inactive rotamer. Next, we determined the structure of OTULIN D336A to 1.35 Å resolution (Figures 2D and S2G and Table S1). There were no global structural perturbations (Figures S2E and S2F), but His339 was now in the active rotamer, and Cys129 showed increased occupancy of the active rotamer (Figure 2D). Consistently, Ub suicide inhibitors that did not modify wild-type (WT) OTULIN modified OTULIN D336A and also OTULIN N341D, in which the catalytic Asn341 was

Figure 2. Structural Analysis of OTULIN

(A) Structure of OTULINcat (aa 80–352). Ub-binding S1 and S1' sites and termini are indicated. The catalytic center is boxed.

(B) Superposition of OTULIN (blue) and OTUB1 (cyan, PDB 2ZFY) (Edelmann et al., 2009).

(C) A close-up image of the OTULIN catalytic triad (Cys129, His339, and Asn341) showing two alternative conformations for His339 and Cys129. Dotted lines indicate hydrogen bonds. A simulated annealing composite omit map (blue, contoured at 1σ) and $|F_o|$-$|F_c|$ map (red, contoured at 3σ) is shown. The active (beige) and inactive (blue) conformation of the catalytic triad are shown. Percentages represent refined occupancies from Refmac5 (Murshudov et al., 2011).

(D) Catalytic center of OTULIN D336A determined at a 1.35 Å resolution (see Figures S2E and S2G) shown as in (C).

(E) OTULIN variants modified by Ub suicide probes, resolved on coomassie-stained SDS-PAGE gels. An 8 kDa shift indicates formation of a covalent OTULIN~Ub complex.

See Figure S1C and the Extended Experimental Procedures.

(F) Structure of OTULIN (blue) in complex with Met1-diUb (with distal Ub in red and proximal Ub in orange), shown in two orientations. The helical arm comprising the S1 and the α1 and α2 helices comprising the S1' Ub-binding sites are labeled, and the catalytic center is indicated.

(G) Surface representation of OTULIN showing S1 (dark red), and S1' (orange) binding sites. Yellow indicates residues interacting with both moieties. Labeled residues were mutated for experiments in (I) and (J).

(H) The structure of Met1-diUb indicating the interfaces with OTULIN colored as in (G). The Ile44 patch (blue) of the distal and the Phe4 patch (cyan; Gln2, Phe4, and Thr14) of the proximal Ub is indicated. Ub $Glu16^{prox}$ is shown in purple.

(I) Met1-diUb hydrolysis assay performed as in Figure 1D with 10 nM OTULIN and OTULIN Ub-binding mutants. Mutations are annotated accordingly: cat, catalytic and Ub-binding sites; S1 and S1', distal and proximal, respectively.

(J) Affinity (K_D) measurements of OTULIN C129A with or without Ub-binding mutations performed with fluorescence anisotropy with FlAsH-tagged Met1-diUb (Ye et al., 2011). Error bars represent SD from the mean of measurements performed in triplicate. WT, wild-type; ND, not determined.

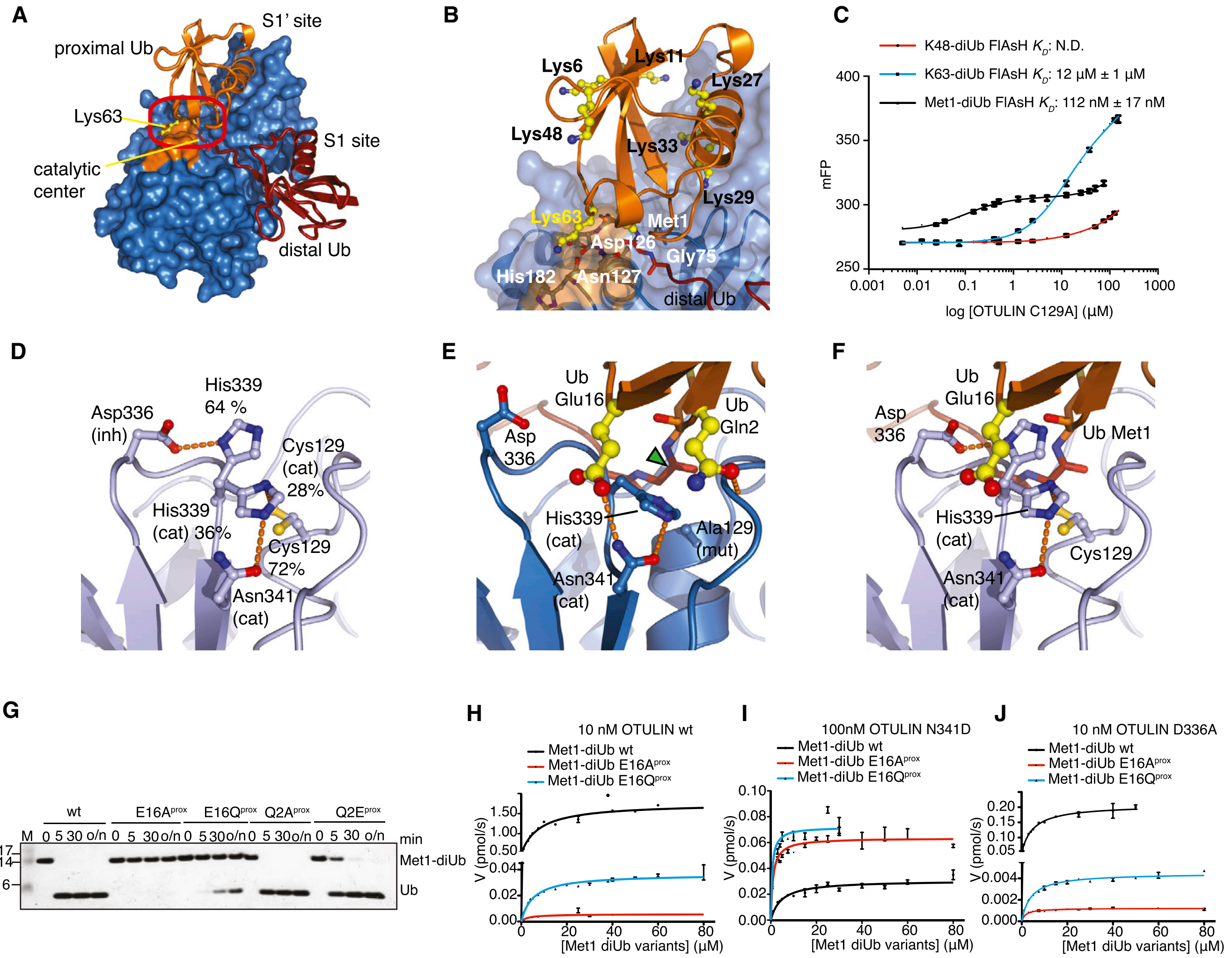

K

	k_{cat} (s^{-1})	K_M (µM)	k_{cat}/K_M ($M^{-1}s^{-1}$) x10³	Fold reduction in enzyme efficiency*
OTULIN wt				
Met1-diUb wt	6.30 ± 0.15	7.98 ± 0.65	789.47	-
Met1-diUb E16A^{prox}	0.026± 0.00051	2.45 ± 0.25	10.61	74
Met1-diUb E16Q^{prox}	0.18 ± 0.0032	4.72 ± 0.34	38.14	21
OTULIN N341D				
Met1-diUb wt	0.014 ± 0.00043	3.71 ± 0.48	3.77	209
Met1-diUb E16A^{prox}	0.032 ± 0.00062	0.99 ± 0.14	32.32	24
Met1-diUb E16Q^{prox}	0.033 ± 0.0011	0.45 ± 0.09	73.33	11
OTULIN D336A				
Met1-diUb wt	0.79 ± 0.031	5.02 ± 0.66	157.37	5
Met1-diUb E16A^{prox}	0.003 ± 0.000056	1.73 ± 0.18	1.73	456
Met1-diUb E16Q^{prox}	0.011 ± 0.00020	4.78 ± 0.36	2.30	343
OTULIN Y91F				
Met1-diUb wt	0.29 ± 0.0079	8.39 ± 0.76	34.56	23

L

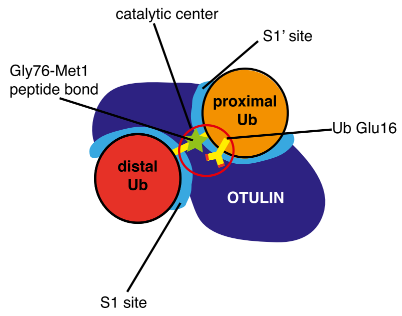

Figure 3. Substrate-Assisted Catalysis in OTULIN

(A) The OTULIN-diUb complex is shown with OTULIN under a blue surface and Met1-diUb colored as red and orange for the distal and proximal moieties, respectively. The catalytic center and Lys63-binding pocket is shown boxed.

(B) A close-up view of (A) showing all Ub Lys residues on the proximal Ub (yellow). Lys63 and Met1 are spatially close.

(C) Affinity measurements of OTULIN C129A with K48-, K63, and Met1-diUb linkages performed with fluorescence anisotropy (Ye et al., 2011). Error bars represent SD from the mean of measurements performed in triplicate.

(D–F) A close-up image of the OTULIN catalytic center showing key residues. Dotted lines indicate hydrogen bonds.

(D) Unliganded OTULIN, shown as in Figure 2C.

(legend continued on next page)

changed to a negatively charged Asp (Figure 2E). Both mutants stabilize His339 in the active conformation, generating a more reactive enzyme.

To understand how OTULIN acted on Met1-polyUb specifically, we determined the structure of OTULINcat C129A bound to Met1-diUb to 1.9 Å resolution (Figures 2F and S2 and Table S1). The distal and proximal Ub moieties occupy extensive S1 and S1' Ub-binding sites on OTULIN, respectively (Figures 2F–2H). Residues mediating Ub binding are highly conserved in OTULIN orthologs (Figure S2I). The binding interface with the distal Ub covers 1,045 $Å^2$ and involves the Ile44 patch that interacts with a helical arm (aa 254–264) conserved in all OTU domains (Figures 2F–2H). However, compared to the OTUB1~Ub structure (Wiener et al., 2012) (Figure S2J), the distal Ub rotates by ~18° in the S1 binding site (Figure S2K). The proximal Ub binds with an interface of 880 $Å^2$ to an S1' Ub-binding site formed by helices α1 and α2 of OTULINcat via an unusual binding surface on Ub involving the Ub helix and the Phe4 patch (Figure 2H). Point mutations in the S1 (L259E and E314R) or S1' (W96A and TQK100-102AAA) Ub-binding sites reduced OTULIN activity toward Met1-diUb (Figure 2I) by decreasing Met1-diUb affinity (Figure 2J).

OTULIN Specificity: Selective diUb Binding

The extensive S1' Ub-binding site is likely to be important for OTULIN specificity in that it orients the proximal Ub such that only Met1 points toward the catalytic center (Figure 3A). In this orientation of the proximal Ub, all Lys residues are remote from the catalytic center, except for Lys63, which is spatially close to Met1 (Figure 3B). Importantly, OTULIN wedges these linkage points apart by two loops (aa 125–127 and 282–284) that fix Lys63 in a dedicated binding pocket (Figure 3B). Nonetheless, a differently linked Ub chain including a Lys63-linked diUb would rotate the proximal Ub moiety by several degrees, and such orientation would likely not fit the OTULIN S1' binding site. Indeed, Lys63-linked diUb bound with 100-fold reduced affinity (<112 nM for Met1-linked diUb versus 12 μM for Lys63-linked diUb), and no binding was detected for Lys48-linked diUb (Figure 3C). This shows that the Ub-binding sites of OTULIN already distinguish between structurally similar Met1 and Lys63 chain types by two orders of magnitude.

OTULIN Specificity: Substrate-Assisted Catalysis

The complex structure revealed that the proximal Ub directly participates in the organization of the catalytic center. Autoinhibition of the catalytic triad in the absence of substrate (Figure 3D) is released by the binding of the Met1-linked proximal Ub (Figures 3E and 3F). The carbonyl group of Met1 sterically interferes with the inhibited conformation of His339, pushing it into an active conformation (Figure 3F). Lys-linked Ub chains lack a structural equivalent of this carbonyl moiety in the linkage (Figure 1A). More significantly, Glu16 of the proximal Ub is inserted into the catalytic center, displacing the inhibitory OTULIN residue Asp336, further restricting the mobility of His339 (Figures 3E and 3F). In addition, Glu16 coordinates the third residue in the catalytic triad Asn341, aligning it toward His339 (Figure 3E).

Importantly, Met1-diUb with mutations of Glu16 in the proximal Ub was hydrolyzed with significantly lower activity in qualitative gel-based assays, whereas mutation of nearby Gln2 (which also interacts with OTULIN, Figure 3E) had no effect (Figures 3G and S3A). All mutants were hydrolyzed by the nonspecific DUB USP21 (Ye et al., 2011) (Figure S3B) and bound to OTULINcat C129A in analytical gel filtration (Figure S3C), and Met1-diUb E16A^{prox} affinity toward OTULIN C129A was only slightly decreased (612 versus <112 nM, Figure S3D).

We used a quantitative fluorescent kinetic assay for diUb cleavage (Virdee et al., 2010) to examine whether Glu16 on a proximal Ub had a direct role in catalysis (Figures 3H and 3I). Met1-diUb E16A^{prox} decreased k_{cat} 240-fold and enzyme efficiency (k_{cat}/K_M) 74-fold (because of a 3.5-fold lower K_M for the mutant) in comparison to Met1-diUb (Figures 3H and 3K). Interestingly, the negative charge on Ub is important for OTULIN activity, given that Met1-diUb E16Q^{prox} still showed a 35-fold lower k_{cat} for WT OTULIN. Next, we tested whether the more reactive OTULIN N341D or D336A mutants (Figure 2E) showed improved diUb hydrolysis activity. WT Met1-diUb is a poor substrate for OTULIN N341D, most likely because of the repulsion of negative charges (Ub Glu16 and OTULIN Asp341) in the catalytic center (Figures 3I and K). Indeed, OTULIN N341D showed improved kinetics when Ub Glu16 was mutated, and both k_{cat} and K_M improved, although WT activity was not regained. The OTULIN N341D mutant worked best with Met1-diUb E16Q^{prox}, suggesting that the requirement for a negative charge was partially compensated (Figures 3J and 3K). The coordination of Asn341 is a key event in OTULIN activation, as was confirmed when the only OTULIN residue coordinating the Asn341 side chain, Tyr91, was mutated to Phe, resulting in 20-fold reduction of k_{cat} while not affecting K_M (Figures 2I, 2J, 3K, and S3F). Strong effects of Glu16 mutation were also observed in the OTULIN D336A mutant (Figures 3J and 3K). Altogether, this showed that the coordination of the catalytic triad through Ub interaction is important for OTULIN activation (Figure 3L).

Hence, we reveal a mechanism of substrate-assisted catalysis in which Glu16 of the proximal Ub activates the catalytic triad of OTULIN by both restricting the movement of the catalytic His339 and introducing a negative charge, presumably for the proper

(E) OTULIN C129A (blue) in complex with Met1-diUb (red and orange). Residues from the proximal Ub are shown in yellow. A green arrow indicates the scissile bond (compare to Figure 1A).

(F) Superposition of (D) with the Met1-diUb from (E). The carbonyl of Met1 and the side chain of Glu16 of the proximal Ub disengage the autoinhibition of His339. Gln2 is omitted for clarity.

(G) OTULIN hydrolysis of Met1-diUb mutated in the proximal moiety performed as in Figure 1D. o/n, overnight incubation.

(H–J) Kinetic parameters of OTULIN variants measured by fluorescence anisotropy. Initial rates of hydrolysis at varying substrate concentrations containing 150 nM FlAsH-tagged Met1-diUb variants were fitted to the Michaelis-Menten kinetic model with GraphPad Prism 5. Error bars represent SDs from the mean of measurements performed in triplicate.

(K) Summary of kinetic parameters measured. *, fold reduction in enzyme efficiency relative to OTULIN WT + Met1-diUb WT.

(L) A schematic representation of OTULIN mechanism, which involved extensive S1 and S1' sites and substrate-assisted catalysis mediated by Ub Glu16.

coordination of Asn341 for catalysis. OTULIN's usage of a catalytic Asn improves interaction with Glu16 containing Met1-linked chains.

It appears that OTULIN has evolved Met1-linkage-specific Ub-binding sites to specifically interact with linear chains. Additionally, to further distinguish chain types, OTULIN invokes a mechanism of substrate-assisted catalysis in order to ensure that only Met1 linkages are hydrolyzed. OTULIN's remarkable specificity suggests that Met1-linked polyUb have to be tightly regulated independently of other ubiquitination events in cells.

Cellular OTULIN Antagonizes LUBAC Signaling

The identification of OTULIN as a Met1-linkage-specific DUB prompted us to study its role in cells. The human *FAM105B* gene is ubiquitously expressed (http://biogps.org/%23goto%3Dgenereport%26id%3D90268). A polyclonal antiserum detected OTULIN in human embryonic kidney (HEK) 293ET and other cell lines (Figure S4A–S4C). C-terminal GFP-tagged OTULIN is cytoplasmic, active, and Met1 specific (Figure S4D–S4F). OTULIN is evolutionarily restricted to vertebrates and selected invertebrate lineages but is not detected in *D. melanogaster* and *C. elegans*. Interestingly, all OTULIN-comprising taxa also contain genes for components of the Met1-polyUb chain assembly machinery LUBAC.

Expression of the LUBAC components HOIP, HOIL-1L, and SHARPIN induced Met1-polyUb, which was removed when OTULIN was coexpressed (Figure 4A, lanes 2 and 3). Inactive OTULIN C129A led to the significant enrichment of Met1-polyUb in cells, and OTULIN Ub chain-binding mutants W96A and L259E (Figures 2I and 2J) increased Met1 linkages in cells, albeit not to the same extent as C129A (Figure 4A, lanes 4–6).

Expression of LUBAC induces NF-κB activation (Gerlach et al., 2011; Ikeda et al., 2011; Tokunaga et al., 2009), which was suppressed when OTULIN was transiently coexpressed (Figure 4B) and also in stable cell lines overexpressing OTULIN (Figures S4G and S4H). Despite the enrichment of Met1-polyUb (Figure 4A), OTULIN catalytic or Ub-binding mutants still inhibited LUBAC-driven NF-κB activity to some extent (Figure 4B). Apparently, the increase in Met1-polyUb alone is not sufficient to augment NF-κB activity (Figures 4A and 4B).

NF-κB activation by TNFα leads to the translocation of the cytosolic p65 NF-κB subunit to the nucleus (Hayden and Ghosh, 2008) (Figures 4C, S5C, and S5D). Transient overexpression of OTULIN or OTULIN C129A blocked p65 nuclear translocation after TNFα stimulation, whereas OTULIN W96A or OTULIN L259E had no effect (Figures 4C, S5C, and S5D). It appears that OTULIN overexpression antagonizes NF-κB activation by removing Met1-polyUb, whereas OTULIN C129A acts as a high-affinity UBD that competes with other Met1-specific UBDs required for NF-κB signaling in a similar manner to the one recently reported for the overexpression of the NEMO UBAN domain (van Wijk et al., 2012).

These results suggested that OTULIN was able to regulate LUBAC mediated processes in cells. One of the few reported targets of LUBAC is NEMO (Tokunaga et al., 2009). Transfection of GST-tagged NEMO resulted in NEMO modification which was prevented in a NEMO K285/309R double mutant where ubiquitination sites are mutated (Tokunaga et al., 2009) (Figure 4D). Co-overexpression of LUBAC resulted in an additional Ub band on NEMO, and the Met1-specific antibody indicated that this additional band was a short Ub chain. This was absent in OTULIN-overexpressing stable cell lines (Figures 4D and S5F) suggesting that OTULIN could remove Met1-polyUb from NEMO.

Glutathione S-transferase (GST) pulldown of NEMO precipitated HOIP, HOIL1, SHARPIN, and OTULIN, suggesting that these proteins might form a complex (Figures 4D and S5F). Immunoprecipitation (IP) of endogenous SHARPIN precipitated endogenous HOIP and, interestingly, also endogenous OTULIN under unstimulated conditions (Figures 4E and S5G). Upon TNFα stimulation, the TNF-R1 was enriched in SHARPIN IPs (Figure 4E), further supporting the idea that a LUBAC-OTULIN complex is formed and that this complex may translocate to the TNF receptor signaling complex (TNF-RSC).

Met1-linked polyUb is known to affect complex assembly at the TNF-RSC (Haas et al., 2009). IP of endogenous NEMO coprecipitates ubiquitinated RIPK1 after TNFα stimulation. Importantly, overexpression of OTULIN abrogated NEMO-RIPK1 complex formation (Figure 4F). This supports the idea that the interaction between NEMO and RIPK1 is stabilized by Met1 linkages and reveals a mechanism of how OTULIN may affect NF-κB activation in response to TNFα stimulation.

Ubiquitin Glu16 Is Important for Met1-polyUb Signaling

Next, with a cellular readout for OTULIN overexpression at hand, we set out to test OTULIN's mechanism of substrate-assisted catalysis in cells by characterizing the effects of an Ub E16A mutant. Control experiments assessing whether this mutant is still assembled in Met1-polyUb by HOIP gave the surprising result that a minimal HOIP ligase construct (Smit et al., 2012; Stieglitz et al., 2012) was impaired in assembling Met1-linked chains from Ub E16A (Figure 5A). Furthermore, fluorescent Met1-diUb E16A^{prox} bound to the NEMO UBAN domain with reduced affinity (Figure 5B), which is consistent with the known interaction between the NEMO UBAN domain and Ub Glu16 (Figure 5C) (Rahighi et al., 2009).

Despite this, co-overexpression of Ub E16A and LUBAC in HEK 293ET cells still activated NF-κB, and this could not be completely reversed by OTULIN (Figure 5D), consistent with OTULIN's inability to efficiently hydrolyze Ub E16A-containing polymers (Figure 3). These results suggested that Ub Glu16 is important in multiple aspects of Met1-polyUb signaling.

OTULIN Affects LUBAC-Mediated Cytokine Responses

Transient overexpression of OTULIN or the well-studied NF-κB inhibitor A20 (Hymowitz and Wertz, 2010) blocks poly(I:C) induced NF-κB activity (Figure 6A). In comparison, OTULIN inhibits TNFα-mediated NF-κB by ~50%, which correlates with effects observed by LUBAC downregulation (Haas et al., 2009; Tokunaga et al., 2009) (Figure 6A). In OTULIN-expressing stable cell lines, transcription of NF-κB target genes was reduced in response to 10 ng/ml TNFα (Figure 6B), and, although IκBα was rapidly phosphorylated and almost completely degraded after 15 min of TNFα stimulation in control cell lines, IκBα phosphorylation, degradation, and NF-κB activation was delayed in

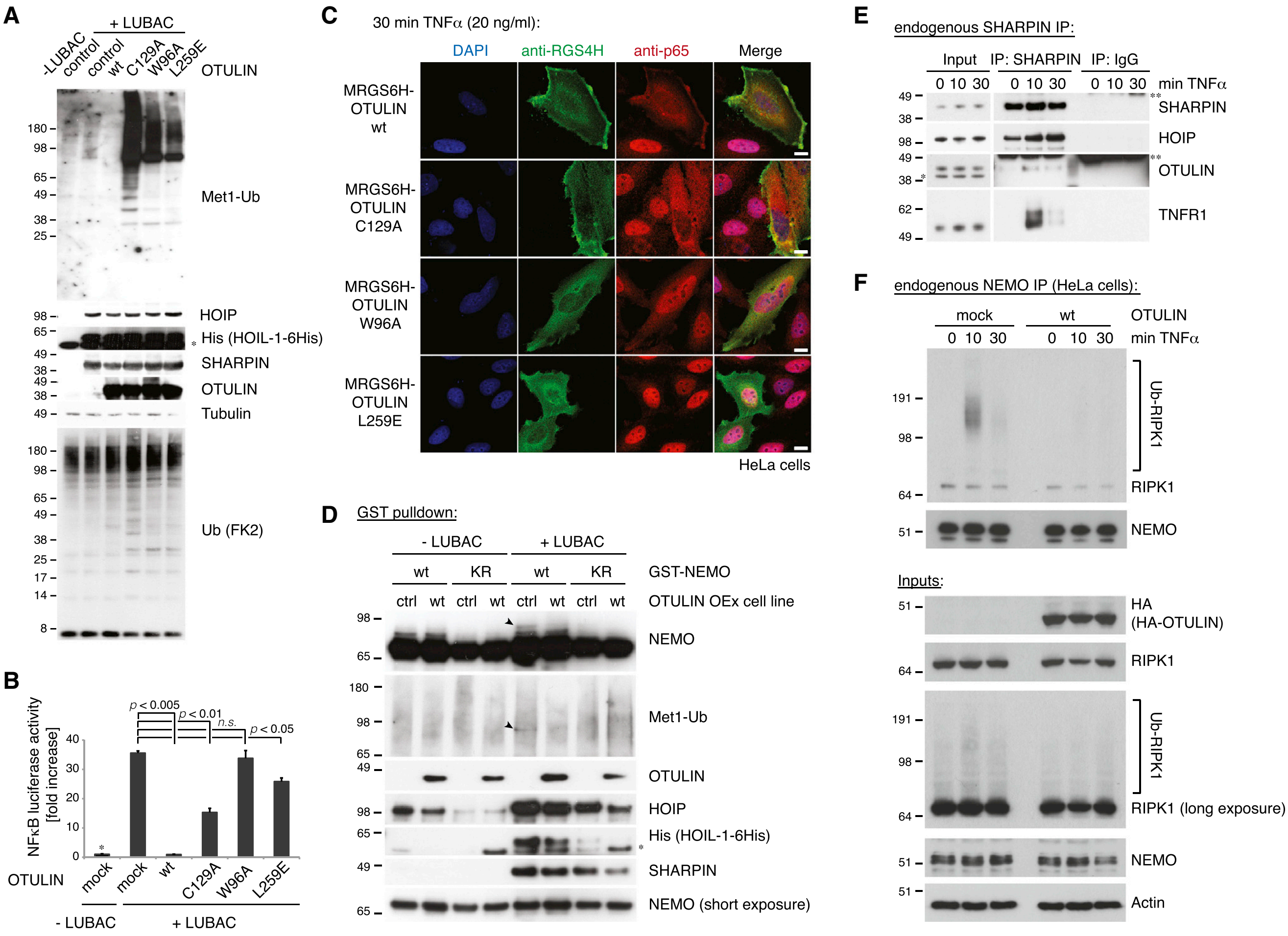

Figure 4. Cellular Functions of OTULIN

(A) HEK 293ET cells were transfected with plasmids for LUBAC and indicated OTULIN variants (see the Extended Experimental Procedures), and lysates were analyzed by western blotting with the indicated antibodies, including the Met1-linkage-specific antibody (Matsumoto et al., 2012).

(B) NF-κB luciferase assays in HEK 293ET cells for the experiment shown in (A). Error bars represent SD from the mean of experiments performed in triplicate. p values are given to indicate significance. *, mean value set to 1; n.s., nonsignificant.

(C) HeLa cells were transiently transfected with indicated plasmids, treated with TNFα (20 ng/ml) for 30 min, and analyzed by immunofluorescence with indicated antibodies (see Figure S5 for controls and quantification). The scale bar represents 10 μm.

(D) Pulldown of GST-tagged NEMO wild-type (WT) or K285/309R (KR), with or without LUBAC, in control or OTULIN-overexpressing T-REx 293 cell lines. Western blotting with indicated antibodies reveals polyUb on NEMO (arrowhead), which is lost when OTULIN is coexpressed.

See also Figure S5.

(E) Immunoprecipitation (IP) of endogenous SHARPIN coprecipitates HOIP and OTULIN and after TNFα stimulation (100 ng/ml), also TNFR1 is shown as revealed by western blotting with the indicated antibodies. *, nonspecific band; **, heavy chain.

See also Figure S5.

(F) IP of endogenous NEMO coprecipitates polyubiquitinated RIPK1 (Ub-RIPK1) after TNFα stimulation (10 ng/ml) of HeLa cells. Western blotting with the indicated antibodies reveals that transient OTULIN overexpression prevents this complex formation.

OTULIN-overexpressing cells (Figure 6C). Moreover, stable overexpression of OTULIN sensitized cells to TNFα-induced cell death (Figures 6D and 6E), consistent with observations in *cpdm* mice that lack Sharpin (Gerlach et al., 2011; Ikeda et al., 2011; Tokunaga et al., 2011; Walczak et al., 2012) or in humans with mutations in HOIL1 (Boisson et al., 2012). OTULIN overexpression promoted enhanced and persistent JNK activation and c-Jun phosphorylation at later time points, leading to the cleavage of PARP and caspase-3, two key players in the regulation of cell-death induction (Figure 6F). As for IκBα phosphorylation, OTULIN overexpression also resulted in a delay of JNK activation kinetics (Figures 6C and 6F).

Small interfering RNA (siRNA) against OTULIN resulted in an increase in LUBAC-induced NF-κB activation in HEK 293ET and U2OS cells and also in T-REx 293 cells stably expressing a doxycycline-inducible OTULIN-targeting microRNA (miRNA) (Figures 7A and S6A–S6C). LUBAC-dependent induction of NF-κB could be prevented by co-overexpression of

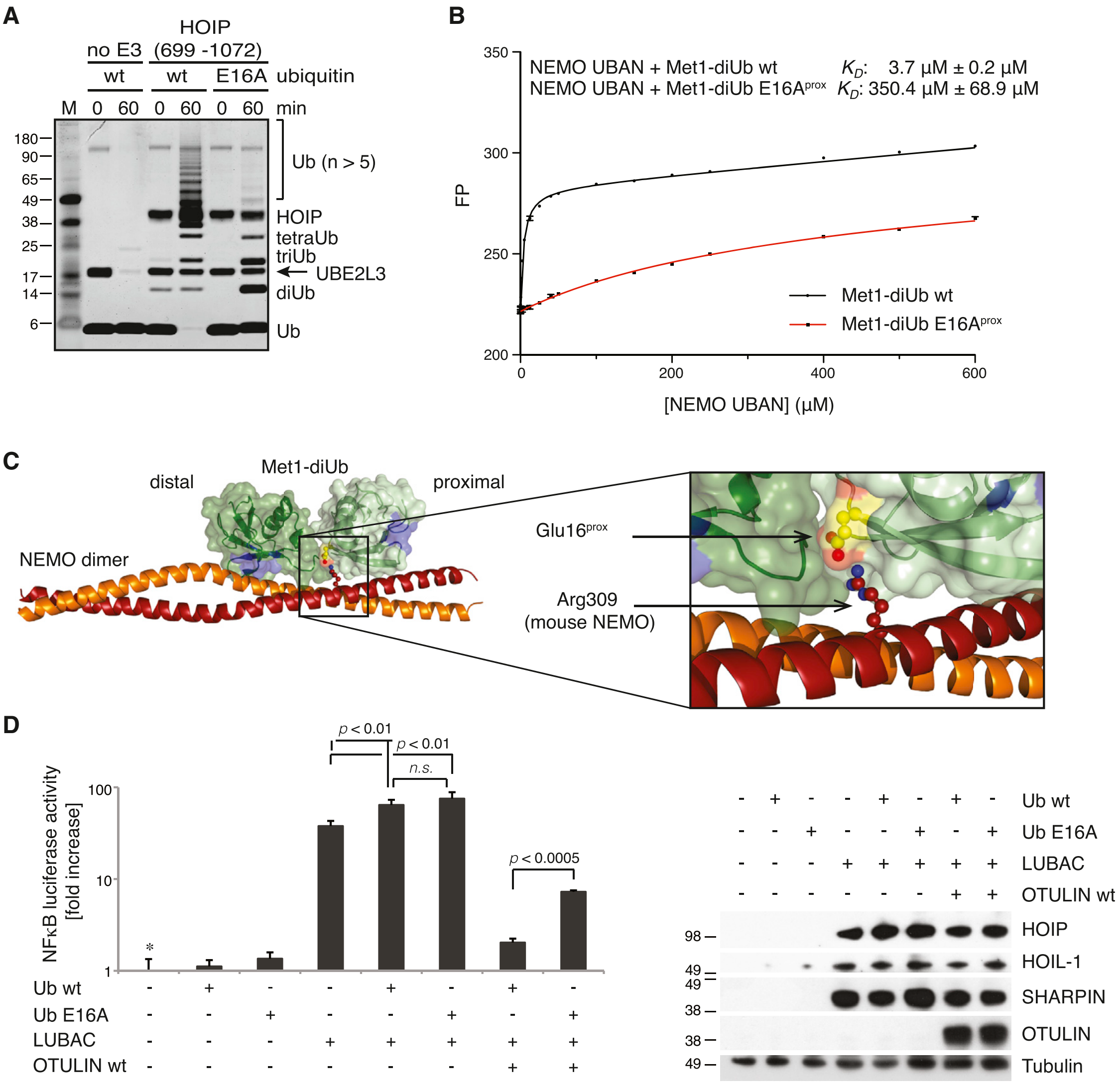

Figure 5. Importance of Ub Glu16 for Met1-polyUb Biology

(A) A minimal HOIP construct (aa 699–1072) that efficiently assembles Met1-Ub chains with WT Ub (Smit et al., 2012; Stieglitz et al., 2012) is less efficient with Ub E16A^{prox} in vitro. A silver-stained SDS-PAGE gel is shown.

(B) Fluorescence anisotropy of NEMO UBAN domain binding to FlAsH-tagged Met1-diUb and Met1-diUb E16A^{prox}. The UBAN domain binds the mutant Ub chain with ~100-fold lower affinity. Error bars represent SD from the mean from triplicate measurements.

(C) Structural basis for decreased affinity of NEMO for Met1-diUb E16A^{prox} mutant. The structure of the NEMO UBAN domain dimer (orange) bound to Met1-diUb is shown (PDB 2ZVN [Rahighi et al., 2009], one diUb omitted for clarity). Ub molecules are shown under a green surface with Ile44 hydrophobic patches in blue. Glu16 and its interacting residue Arg309 (mouse NEMO, corresponding human residue Arg312) are shown in stick representation. The inset highlights this interaction. Glu16^{prox} also bridges the two Ub moieties and interacts with the C terminus of a distal Ub (data not shown).

(D) Luciferase assays performed as in Figure 4B for HEK 293ET cells transfected with or without LUBAC, WT OTULIN, and Ub WT or Ub E16A. p values are given to indicate significance. *, mean value set to 1; n.s., nonsignificant. Input levels of transfected proteins, analyzed by western blotting with the indicated antibodies, are shown on the right.

active, but not inactive, OTULIN (Figure 7A). Interestingly, western blotting against the overexpressed LUBAC components revealed that HOIP was ubiquitinated in OTULIN miRNA cell lines (Figure 7B), and coexpression of OTULIN removed these chains, showing that they are Met1 linked (Figure 7B). This suggested that one role of OTULIN in the LUBAC complex (Figure 4E) is to prevent the autoubiquitination of HOIP. However, LUBAC recruitment to the TNF-RSC was unchanged in OTULIN knockdown cell lines, and Met1-Ub linkages were enriched in the TNF-RSC, implying that OTULIN also regulates Met1-Ub on other components (Figure 7C). Accordingly, we observed a slight increase in RIPK1 ubiquitination enriched with a Met1-linkage-specific Ub-binding matrix in OTULIN-depleted U2OS cells (Figure 7D), suggesting that RIPK1 Met1 ubiquitination in response to TNFα is targeted by OTULIN (Figure 4F).

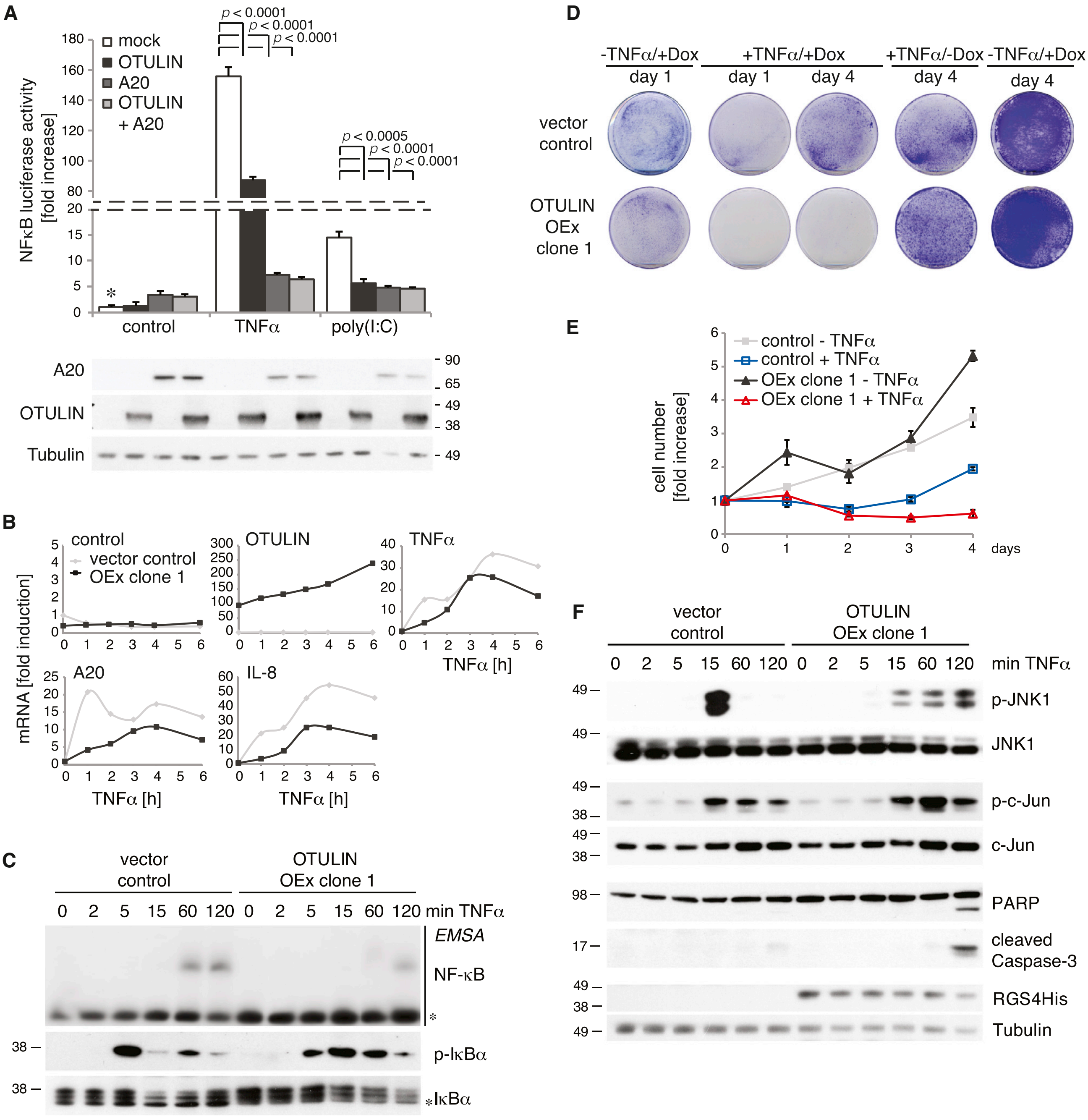

Figure 6. OTULIN Overexpression Inhibits TNFα Signaling

(A) Luciferase assays performed as in Figure 4B in HEK 293ET cells transfected with OTULIN, A20, or both OTULIN and A20 and stimulated with TNFα (100 ng/ml) or poly(I:C) (10 μg/ml). Western blots below show transfected protein levels. Error bars represent SD from the mean for experiments performed in triplicate. p values are given to indicate significance. *, the control set to 1.

(B–F) Stable doxycycline-inducible control and OTULIN-overexpressing T-REx 293 cells after treatment with 1 μg/ml doxycyclin (Dox) for 24 hr (see the Extended Experimental Procedures).

(B) Quantitative PCR (qPCR) analysis of selected NF-κB target genes in control (gray) and OTULIN-overexpressing cells (black) after treatment with 10 ng/ml TNFα for the indicated times.

(C) Analysis of TNFα-stimulated NF-κB activation and IκBα phosphorylation upon TNFα treatment (100 ng/ml) over the indicated time course by western blotting with indicated antibodies and EMSA. See (F) for tubulin control.

(D) Clonogenic survival of indicated cell lines ± Dox and ± TNFα (50 ng/ml) for 24 hr (see the Extended Experimental Procedures).

(E) Cell viability counts of cells treated as in (D). Error bars represent SD from the mean for experiments performed in triplicate.

(F) Analysis of TNFα-stimulated signaling cascades upon TNFα treatment (100 ng/ml) over the indicated time course by western blotting.

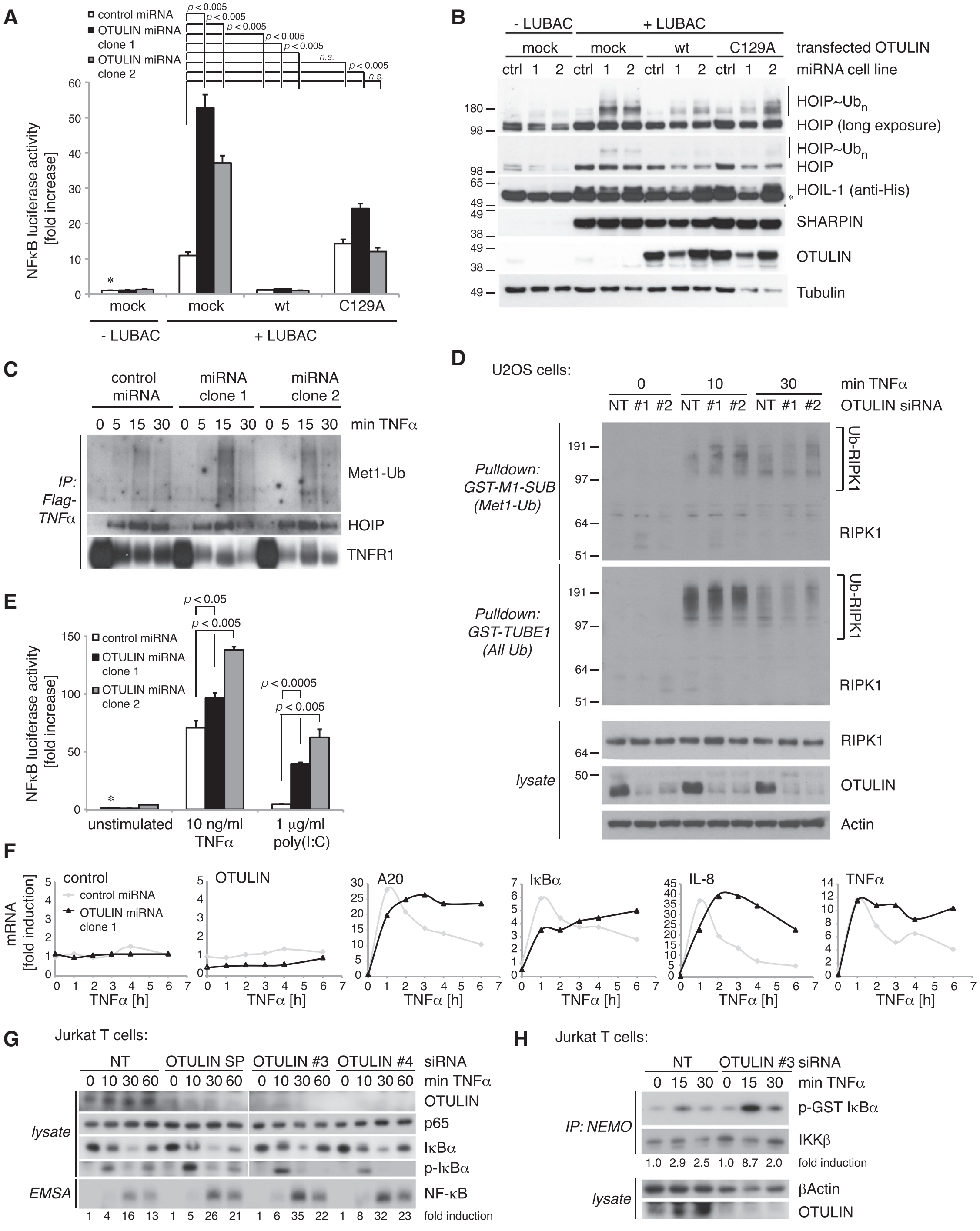

Figure 7. OTULIN Depletion Amplifies LUBAC Signaling

(A–C, E–F) Experiments in stable T-REx 293 cell lines inducibly expressing control or OTULIN-targeting miRNA (see Experimental Procedures and Figure S6).

(A) NF-κB luciferase activity shown as in Figure 4B in cells transfected with LUBAC and indicated OTULIN variants. Error bars represent SD from the mean of experiments performed in duplicate. p values are given to indicate significance. *, the control set to 1; n.s., nonsignificant.

(B) Western blotting analysis of lysates from (A) with indicated antibodies. *, nonspecific band.

(C) Immunoprecipitation of the TNF-RSC by FLAG-TNFα (100 ng/ml) from control and OTULIN-depleted cell lines at indicated time points western blotted against Met1-polyUb, HOIP, and TNFR1.

(D) Met1-Ub-specific GST-M1-SUB or general GST-TUBE1 Ub pulldown from U2OS cells stimulated with TNFα (10 ng/ml) after transfection of OTULIN siRNA or a nontargeting (NT) control siRNA.

(legend continued on next page)

Stable OTULIN knockdown affected cytokine responses and led to enhanced NF-κB activation after poly(I:C) and TNFα (Figure 7E), and, even though the initial induction of NF-κB target genes was not severely altered after OTULIN knockdown, expression of A20, IκBα, IL-8, and TNFα was sustained in response to TNFα (Figure 7F). Initial phosphorylation and degradation of IκBα after TNFα stimulation was only slightly enhanced in OTULIN knockdown cells (Figure S6D). However, JNK and c-Jun phosphorylation was sustained at late time points, again leading to cell death (Figures S6D–S6F). The fact that both overexpression and knockdown of OTULIN led to cell death was surprising and requires further investigation.

OTULIN knockdown cell lines were deregulated in their NF-κB response, but we could not observe a strong effect on canonical NF-κB signaling. Jurkat cells express higher amounts of OTULIN (Figure S4C), and OTULIN knockdown by siRNA led to an increase in phospho-IκBα and subsequent enhancement of NF-κB DNA binding (Figure 7G). This was due to increased IKK activation (Figure 7H), consistent with current models of LUBAC function (Tokunaga and Iwai, 2012; Walczak et al., 2012). Altogether, our data are consistent with a role of OTULIN in LUBAC-mediated Met1-polyUb signaling.

DISCUSSION

Here, we identify FAM105B/OTULIN as a human OTU DUB specific for Met1-linked Ub chains. OTULIN is a missing piece in Met1-Ub chain biology, for which no linkage-specific DUB has been described to date. Our data suggest that OTULIN is an antagonist of LUBAC in vitro and in cells. We reveal the molecular basis for the remarkable Met1 linkage specificity by structural and biophysical studies and give insights into potential roles of OTULIN as a LUBAC interactor and antagonist in cells. Consistent with the biochemical findings, overexpression of OTULIN removes Met1-polyUb and dampens LUBAC-mediated NF-κB signaling, whereas knockdown of OTULIN leads to increased Met1-polyUb, IKK activation, and NF-κB signaling after TNFα stimulation. However, both overexpression and knockdown of OTULIN sensitized cells to TNFα-induced cell death, suggesting more complex roles for Met1 linkages in balancing signaling pathways downstream of the TNFα receptor.

Structural Insights into Ub Chain Linkage Specificity in DUBs

Linkage specificity in DUBs is not well understood, and the structure of apo OTULIN did not explain its Met1 specificity, because of its structural similarity to the Lys48-specific OTUB1. The key insight into linkage specificity for these two enzymes came from the complex structures of the enzyme with two Ub molecules bound across the active site.

A high-affinity Ub-binding site in OTULIN allows it to select Met1-linked chains with 100-fold preference over Lys63 linkages and disallows the binding of Lys48-linked and presumably other chains. This mechanism is shared by many DUBs; e.g., TRABID (Licchesi et al., 2012) and OTUB1 (Juang et al., 2012; Wiener et al., 2012). However, in a physiological situation where multiple Ub chain linkages are often mixed, substrate-targeted DUBs may cleave additional Ub chain types. OTULIN prevents such promiscuity by requiring a properly positioned Ub residue, Glu16, for catalytic efficiency. Even in Lys63-linked chains, the required rotation of the proximal Ub would displace Ub Glu16, preventing activity. Therefore, OTULIN is the first DUB for which substrate-assisted catalysis has been demonstrated to achieve linkage specificity. It is possible that substrate-assisted catalysis explains the linkage (or substrate) specificity of other DUBs. It will also be interesting to see whether DUB activators exist that complement the catalytic triad in an analogous manner as that observed in OTULIN.

Ub-mediated substrate-assisted catalysis was recently shown to be crucial for the assembly of Lys11-linked polyUb, where Ub Glu34 complements the active site of the E2 Ub-conjugating enzyme UBE2S (Wickliffe et al., 2011). The fact that Glu16 also impairs HOIP activity also suggests the functionality of this side chain in assembly reactions. Altogether, this shows that Ub is more than just a binding partner for other proteins and that it can actively participate in enzymatic reactions.

Cellular Role of OTULIN in Counteracting LUBAC

In transfection experiments, we demonstrated that OTULIN could counteract cellular LUBAC responses, suggesting that OTULIN helps to balance the amount of Met1-polyUb in cells. Furthermore, the mechanism of substrate-assisted catalysis suggests that OTULIN may not target individual proteins but, instead, target Met1-linked Ub chains, regardless of where they are attached. Indeed, we identified three proteins that change in their ubiquitination status when OTULIN levels are modulated. NEMO and RIPK1 are among the few reported targets of LUBAC (Gerlach et al., 2011; Tokunaga et al., 2009).

We provide evidence that OTULIN directly interacts with LUBAC. The observation that depletion of OTULIN leads to the modification of HOIP with Met1-polyUb suggests that HOIP, like many other E3 ligases, undergoes autoubiquitination and that OTULIN has the ability to prevent this. This most likely explains why the overexpression of OTULIN chain-binding mutants lead to increased Met1-polyUb in cells (Figure 4A). Moreover, this interaction most likely recruits OTULIN to LUBAC targets. However, the functional consequences of a LUBAC-OTULIN interaction are unclear, given that HOIP stability, recruitment, and activity appear to be unaffected, and this requires further investigation.

(E) NF-κB luciferase activity in control and OTULIN-depleted cell lines after treatment with TNFα (10 ng/ml) or poly(I:C) (1 μg/ml). Error bars represent SD from the mean of experiments performed in duplicate. p values are given to indicate significance. *, the control set to 1; n.s., nonsignificant.

(F) Analysis of selected NF-κB target genes by qPCR in control and OTULIN-depleted cell lines over a time course of TNFα stimulation (10 ng/ml).

(G) Jurkat cells transfected with nontargeting (NT) control siRNA or three different OTULIN-specific siRNAs were treated with TNFα (25 ng/ml) as indicated. EMSA signals were quantified by densitometry. SP, smart pool.

(H) OTULIN or control siRNA-transfected Jurkat cells were stimulated for 15 and 30 min with TNFα (25 ng/ml). After NEMO IP, kinase assays were performed with GST-IκBα (aa 1–53) as a substrate and quantified by densitometry.

Regarding the functional requirement for Met1-linked chains in NF-κB responses, key questions remain to be answered. First, in contrast to Lys63-linked chains, for which roles in NF-κB signaling have long been verified, for example, by elegant replacement strategies (Xu et al., 2009), Met1-linked polymers have a very low abundance, and are hard to detect in cell lysates. This may change with the discovery that catalytically inactive OTULIN (Figure 4A) or OTULIN knockdown (Figure 7C) leads to the stabilization of Met1 linkages. A second, more important issue is the lack of proteins modified with Met1-polyUb in vivo after a physiological stimulus. We believe that OTULIN can also be instrumental here, given that its specificity can be exploited as a Met1-specific Ub-binding protein when inactivated or in mass spectrometry methods. We anticipate that the identification of LUBAC and OTULIN targets may reveal a surprising variety of cellular proteins not restricted to NF-κB signaling.

Role of OTULIN in Cytokine Responses

Our data identify roles of OTULIN in TNFα signaling, which is in agreement with reported roles of Met1-polyUb in this pathway (Haas et al., 2009; Tokunaga et al., 2009; Walczak et al., 2012). However, in TNFα signaling, the putative redundant or nonredundant involvement of many different Ub chain types leads to a complicated interplay of Ub signaling (Kulathu and Komander, 2012). The functional importance of Met1-linked chains in the mix is supported by genetic evidence from *cpdm* mice lacking SHARPIN (Gerlach et al., 2011; Ikeda et al., 2011; Tokunaga et al., 2011) and data from human patients with mutations in HOIL1 (Boisson et al., 2012). So far, there is no unifying molecular mechanism that explains why so many different forms of polyUb are seemingly important for the activation of identical kinase cascades upon TNFα stimulation. Interestingly, the effects of OTULIN overexpression or knockdown seem more pronounced in poly(I:C) versus TNFα signaling (Figures 6A and 7E), and it will be important to study roles of OTULIN in other pathways that depend on Met1-linked chains, such as NOD2 signaling (Damgaard et al., 2012).

Our data support a role of Met1 linkages in providing an important scaffold for productive complex formation, given that the loss of Met1-linked chains induced by OTULIN overexpression prevents the association of NEMO with ubiquitinated RIPK1. Ubiquitinated RIPK1 is targeted also by the DUBs A20 (Hymowitz and Wertz, 2010) and CYLD (Sun, 2010). CYLD hydrolyzes Met1 linkages (Komander et al., 2009) and, hence, could also contribute to antagonize LUBAC signaling. However, although A20 and CYLD are induced by TNFα for the establishment of a negative feedback loop, neither OTULIN (Figure 7F) nor LUBAC (Haas et al., 2009) are induced by TNFα, again suggesting that they function as a differentially regulated signaling module. OTULIN is subject to phosphorylation, acetylation, and ubiquitination in cells, and it will be interesting to study whether these modifications regulate its activity or function.

Overall, our data suggest that the identified DUB OTULIN is an antagonist of the LUBAC E3 ligase complex. Consistent with reported roles for Met1-polyUb, changing OTULIN expression affects LUBAC-mediated processes, including NF-κB signaling. Detailed genetic analysis will be necessary in order to understand how LUBAC and OTULIN balance Met1-polyUb chains in vivo. This might confirm some reported roles and may reveal new cellular roles for this atypical Ub chain type.

EXPERIMENTAL PROCEDURES

Identification of OTULIN

FAM105B/OUTLIN was identified bioinformatically with generalized profile analysis, as described in the Extended Experimental Procedures.

Cloning, Expression, and Purification

FAM105B was cloned from brain complementary DNA (Invitrogen), expressed in *E. coli* from pOPIN-F vector, and purified by anion exchange and gel filtration.

Crystallization and Structure Determination

Apo, SeMet-substituted, and mutant OTULIN were crystalized from 100 mM MES and imidazole, 30 mM $MgCl_2$, 30 mM $CaCl_2$, 10% (w/v) PEG 4k, and 20% glycerol (pH 6.5). The structure of apo OTULIN was determined by single anomalous dispersion, and subsequent structures were determined by molecular replacement.

Qualitative DUB Linkage Specificity Assay

Qualitative deubiquitination assays were performed as previously described (Komander et al., 2009).

Binding Studies and DUB Kinetics

Binding studies were performed as in Ye et al. (2011), and kinetic studies were performed as in Virdee et al. (2010) with FlAsH-tagged Met1-diUb variants.

Cellular Studies of OTULIN

OTULIN and A20 were expressed from pOPIN-F or pcDNA4/TO/MRGS6H, HOIP, SHARPIN and HOIL-1L from pcDNA3.1, and GST-tagged NEMO were expressed from pEBG vectors. Stable T-REx 293 cell lines overexpressing pcDNA4/TO/MRGS6H-OTULIN or a pDEST30-EmGFP construct with an miRNA targeting the 3′ untranslated region of *FAM105B* were generated according to the manufacturer's protocol (Invitrogen). Knockdown analysis was performed in miRNA cell lines or with eight different siRNA sequences, as listed in the Extended Experimental Procedures.

NF-κB Activity Analysis

NF-κB activity was assessed by luciferase assays, for which cells were cotransfected with M3P sin rev κB firefly and pRL-TK *Renilla* (Promega) luciferase plasmids by immunofluorescence with anti-p65 staining or by quantitative PCR, as described in the Extended Experimental Procedures. TNFα signaling was analyzed by western blotting with the antibodies listed in the Extended Experimental Procedures.

ACCESSION NUMBERS

Coordinates and structure factors have been deposited in the Protein Data Bank and can be found at accession numbers 3ZNV (WT OTULIN apo), 3ZNX (OTULIN apo D336A), and 3ZNZ (OTULIN-Met1-diUb).

SUPPLEMENTAL INFORMATION

Supplemental Information includes Extended Experimental Procedures, six figures, and one table and can be found with this article online at http://dx.doi.org/10.1016/j.cell.2013.05.014.

ACKNOWLEDGMENTS

We would like to thank S. McLaughlin, R. Williams, A. Finch, F. Randow, G. Murshudov (Medical Research Council [MRC] Laboratory of Molecular Biology), G. Praefcke (University of Cologne), and members of the D. Komander lab for advice, reagents, and critical comments on the manuscript.

Liposome reagent for Jurkat T cell transfection was a kind gift of Silence Therapeutics, Berlin. This work was supported by the MRC (U105192732 to D. Komander), the European Research Council (309756 to D. Komander), the European Molecular Biology Organization Young Investigator program (D.K.), the Lister Institute for Preventive Medicine (D. Komander), the German Research Foundation (DFG Priority Programme SPP1365 to K.H. and D. Krappmann), the Novo Nordisk Foundation (M.G.H.), and a Steno Fellowship from the Danish Council for Independent Research (M.G.H.). Crystallographic data were collected at the European Synchrotron Radiation Facility at beam lines ID14-4 and ID23-1 and at Diamond Light Source beam line I04-1.

Received: March 1, 2013
Revised: April 3, 2013
Accepted: May 6, 2013
Published: June 6, 2013

REFERENCES

Amerik AYu, Swaminathan, S., Krantz, B.A., Wilkinson, K.D., and Hochstrasser, M. (1997). In vivo disassembly of free polyubiquitin chains by yeast Ubp14 modulates rates of protein degradation by the proteasome. EMBO J. *16*, 4826–4838.

Behrends, C., and Harper, J.W. (2011). Constructing and decoding unconventional ubiquitin chains. Nat. Struct. Mol. Biol. *18*, 520–528.

Boisson, B., Laplantine, E., Prando, C., Giliani, S., Israelsson, E., Xu, Z., Abhyankar, A., Israël, L., Trevejo-Nunez, G., Bogunovic, D., et al. (2012). Immunodeficiency, autoinflammation and amylopectinosis in humans with inherited HOIL-1 and LUBAC deficiency. Nat. Immunol. *13*, 1178–1186.

Borodovsky, A., Ovaa, H., Kolli, N., Gan-Erdene, T., Wilkinson, K.D., Ploegh, H.L., and Kessler, B.M. (2002). Chemistry-based functional proteomics reveals novel members of the deubiquitinating enzyme family. Chem. Biol. *9*, 1149–1159.

Bucher, P., Karplus, K., Moeri, N., and Hofmann, K. (1996). A flexible motif search technique based on generalized profiles. Comput. Chem. *20*, 3–23.

Damgaard, R.B., Nachbur, U., Yabal, M., Wong, W.W.-L., Fiil, B.K., Kastirr, M., Rieser, E., Rickard, J.A., Bankovacki, A., Peschel, C., et al. (2012). The ubiquitin ligase XIAP recruits LUBAC for NOD2 signaling in inflammation and innate immunity. Mol. Cell *46*, 746–758.

Edelmann, M.J., Iphöfer, A., Akutsu, M., Altun, M., di Gleria, K., Kramer, H.B., Fiebiger, E., Dhe-Paganon, S., and Kessler, B.M. (2009). Structural basis and specificity of human otubain 1-mediated deubiquitination. Biochem. J. *418*, 379–390.

Faesen, A.C., Luna-Vargas, M.P.A., Geurink, P.P., Clerici, M., Merkx, R., van Dijk, W.J., Hameed, D.S., El Oualid, F., Ovaa, H., and Sixma, T.K. (2011). The differential modulation of USP activity by internal regulatory domains, interactors and eight ubiquitin chain types. Chem. Biol. *18*, 1550–1561.

Frias-Staheli, N., Giannakopoulos, N.V., Kikkert, M., Taylor, S.L., Bridgen, A., Paragas, J., Richt, J.A., Rowland, R.R., Schmaljohn, C.S., Lenschow, D.J., et al. (2007). Ovarian tumor domain-containing viral proteases evade ubiquitin- and ISG15-dependent innate immune responses. Cell Host Microbe *2*, 404–416.

Gerlach, B., Cordier, S.M., Schmukle, A.C., Emmerich, C.H., Rieser, E., Haas, T.L., Webb, A.I., Rickard, J.A., Anderton, H., Wong, W.W.-L., et al. (2011). Linear ubiquitination prevents inflammation and regulates immune signalling. Nature *471*, 591–596.

Haas, T.L., Emmerich, C.H., Gerlach, B., Schmukle, A.C., Cordier, S.M., Rieser, E., Feltham, R., Vince, J., Warnken, U., Wenger, T., et al. (2009). Recruitment of the linear ubiquitin chain assembly complex stabilizes the TNF-R1 signaling complex and is required for TNF-mediated gene induction. Mol. Cell *36*, 831–844.

Hayden, M.S., and Ghosh, S. (2008). Shared principles in NF-kappaB signaling. Cell *132*, 344–362.

Hershko, A., and Ciechanover, A. (1998). The ubiquitin system. Annu. Rev. Biochem. *67*, 425–479.

Hymowitz, S.G., and Wertz, I.E. (2010). A20: from ubiquitin editing to tumour suppression. Nat. Rev. Cancer *10*, 332–341.

Ikeda, F., Deribe, Y.L., Skånland, S.S., Stieglitz, B., Grabbe, C., Franz-Wachtel, M., van Wijk, S.J.L., Goswami, P., Nagy, V., Terzic, J., et al. (2011). SHARPIN forms a linear ubiquitin ligase complex regulating NF-κB activity and apoptosis. Nature *471*, 637–641.

Juang, Y.-C., Landry, M.-C., Sanches, M., Vittal, V., Leung, C.C.Y., Ceccarelli, D.F., Mateo, A.-R.F., Pruneda, J.N., Mao, D.Y.L., Szilard, R.K., et al. (2012). OTUB1 co-opts Lys48-linked ubiquitin recognition to suppress E2 enzyme function. Mol. Cell *45*, 384–397.

Karin, M., and Ben-Neriah, Y. (2000). Phosphorylation meets ubiquitination: the control of NF-[kappa]B activity. Annu. Rev. Immunol. *18*, 621–663.

Kayagaki, N., Phung, Q., Chan, S., Chaudhari, R., Quan, C., O'Rourke, K.M., Eby, M., Pietras, E., Cheng, G., Bazan, J.F., et al. (2007). DUBA: a deubiquitinase that regulates type I interferon production. Science *318*, 1628–1632.

Kirisako, T., Kamei, K., Murata, S., Kato, M., Fukumoto, H., Kanie, M., Sano, S., Tokunaga, F., Tanaka, K., and Iwai, K. (2006). A ubiquitin ligase complex assembles linear polyubiquitin chains. EMBO J. *25*, 4877–4887.

Komander, D., and Rape, M. (2012). The ubiquitin code. Annu. Rev. Biochem. *81*, 203–229.

Komander, D., Reyes-Turcu, F., Licchesi, J.D.F., Odenwaelder, P., Wilkinson, K.D., and Barford, D. (2009). Molecular discrimination of structurally equivalent Lys 63-linked and linear polyubiquitin chains. EMBO Rep. *10*, 466–473.

Kulathu, Y., and Komander, D. (2012). Atypical ubiquitylation - the unexplored world of polyubiquitin beyond Lys48 and Lys63 linkages. Nat. Rev. Mol. Cell Biol. *13*, 508–523.

Licchesi, J.D.F., Mieszczanek, J., Mevissen, T.E.T., Rutherford, T.J., Akutsu, M., Virdee, S., El Oualid, F., Chin, J.W., Ovaa, H., Bienz, M., and Komander, D. (2012). An ankyrin-repeat ubiquitin-binding domain determines TRABID's specificity for atypical ubiquitin chains. Nat. Struct. Mol. Biol. *19*, 62–71.

Matsumoto, M.L., Dong, K.C., Yu, C., Phu, L., Gao, X., Hannoush, R.N., Hymowitz, S.G., Kirkpatrick, D.S., Dixit, V.M., and Kelley, R.F. (2012). Engineering and structural characterization of a linear polyubiquitin-specific antibody. J. Mol. Biol. *418*, 134–144.

Mevissen, T.E., Hospenthal, M.K., Geurink, P.P., Elliott, P.R., Akutsu, M., Arnaudo, N., Ekkebus, R., Kulathu, Y., Wauer, T., El Oualid, F., et al. (2013). OTU Deubiquitinases Reveal Mechanisms of Linkage Specificity and Enable Ubiquitin Chain Restriction Analysis. Cell *154*. Published online July 3, 2013. 10.1016/j.cell.2013.05.046.

Murshudov, G.N., Skubák, P., Lebedev, A.A., Pannu, N.S., Steiner, R.A., Nicholls, R.A., Winn, M.D., Long, F., and Vagin, A.A. (2011). REFMAC5 for the refinement of macromolecular crystal structures. Acta Crystallogr. D Biol. Crystallogr. *67*, 355–367.

Nakada, S., Tai, I., Panier, S., Al-Hakim, A., Iemura, S.-I., Juang, Y.-C., O'Donnell, L., Kumakubo, A., Munro, M., Sicheri, F., et al. (2010). Non-canonical inhibition of DNA damage-dependent ubiquitination by OTUB1. Nature *466*, 941–946.

Ozkaynak, E., Finley, D., and Varshavsky, A. (1984). The yeast ubiquitin gene: head-to-tail repeats encoding a polyubiquitin precursor protein. Nature *312*, 663–666.

Rahighi, S., Ikeda, F., Kawasaki, M., Akutsu, M., Suzuki, N., Kato, R., Kensche, T., Uejima, T., Bloor, S., Komander, D., et al. (2009). Specific recognition of linear ubiquitin chains by NEMO is important for NF-kappaB activation. Cell *136*, 1098–1109.

Smit, J.J., Monteferrario, D., Noordermeer, S.M., van Dijk, W.J., van der Reijden, B.A., and Sixma, T.K. (2012). The E3 ligase HOIP specifies linear ubiquitin chain assembly through its RING-IBR-RING domain and the unique LDD extension. EMBO J. *31*, 3833–3844.

Stieglitz, B., Morris-Davies, A.C., Koliopoulos, M.G., Christodoulou, E., and Rittinger, K. (2012). LUBAC synthesizes linear ubiquitin chains via a thioester intermediate. EMBO Rep. *13*, 840–846.

Sun, S.-C. (2010). CYLD: a tumor suppressor deubiquitinase regulating NF-kappaB activation and diverse biological processes. Cell Death Differ. *17*, 25–34.

Tokunaga, F., and Iwai, K. (2012). LUBAC, a novel ubiquitin ligase for linear ubiquitination, is crucial for inflammation and immune responses. Microbes Infect. *14*, 563–572.

Tokunaga, F., Sakata, S.-I., Saeki, Y., Satomi, Y., Kirisako, T., Kamei, K., Nakagawa, T., Kato, M., Murata, S., Yamaoka, S., et al. (2009). Involvement of linear polyubiquitylation of NEMO in NF-kappaB activation. Nat. Cell Biol. *11*, 123–132.

Tokunaga, F., Nakagawa, T., Nakahara, M., Saeki, Y., Taniguchi, M., Sakata, S.-I., Tanaka, K., Nakano, H., and Iwai, K. (2011). SHARPIN is a component of the NF-κB-activating linear ubiquitin chain assembly complex. Nature *471*, 633–636.

van Wijk, S.J.L., Fiskin, E., Putyrski, M., Pampaloni, F., Hou, J., Wild, P., Kensche, T., Grecco, H.E., Bastiaens, P., and Dikic, I. (2012). Fluorescence-based sensors to monitor localization and functions of linear and K63-linked ubiquitin chains in cells. Mol. Cell *47*, 797–809.

Virdee, S., Ye, Y., Nguyen, D.P., Komander, D., and Chin, J.W. (2010). Engineered diubiquitin synthesis reveals Lys29-isopeptide specificity of an OTU deubiquitinase. Nat. Chem. Biol. *6*, 750–757.

Walczak, H., Iwai, K., and Dikic, I. (2012). Generation and physiological roles of linear ubiquitin chains. BMC Biol. *10*, 23.

Wickliffe, K.E., Lorenz, S., Wemmer, D.E., Kuriyan, J., and Rape, M. (2011). The mechanism of linkage-specific ubiquitin chain elongation by a single-subunit E2. Cell *144*, 769–781.

Wiener, R., Zhang, X., Wang, T., and Wolberger, C. (2012). The mechanism of OTUB1-mediated inhibition of ubiquitination. Nature *483*, 618–622.

Xu, M., Skaug, B., Zeng, W., and Chen, Z.J. (2009). A ubiquitin replacement strategy in human cells reveals distinct mechanisms of IKK activation by TNFalpha and IL-1beta. Mol. Cell *36*, 302–314.

Ye, Y., Akutsu, M., Reyes-Turcu, F., Enchev, R.I., Wilkinson, K.D., and Komander, D. (2011). Polyubiquitin binding and cross-reactivity in the USP domain deubiquitinase USP21. EMBO Rep. *12*, 350–357.

Cell

EXTENDED EXPERIMENTAL PROCEDURES

Bioinformatics

Multiple sequence alignments were created by using the L-INS-I algorithm of the MAFFT package (Katoh et al., 2002). Sequence profiles were constructed, scaled, and subjected to iterative refinement using programs from the PFTOOLS package (Bucher et al., 1996). Only database hits with *p-value*s < 0.01 were included in the next iteration cycle. The significance of novel OTU assignments was further assessed by HMM-HMM comparison using the HHSEARCH program (Söding, 2005).

Materials and Reagents

Di- and tetraUb for Lys6, Lys11, Lys48, Lys63 were generated using published procedures (Bremm et al., 2010; Hospenthal et al., 2013; Komander et al., 2009). Met1-linked di- and tetraUb was produced according to (Komander et al., 2009). DiUb of Lys27-, Lys29- and Lys33-linkages were purchased from Boston Biochem. Proteins and epitope tags were detected with the following antibodies: anti-A20 (59A426) (Imgenex); anti-Caspase-3 (#9662), anti-c-Jun (60A8), anti-phospho-IκBα (14D4 or 5A5), anti-iκBα (L35A5), anti-JNK1 (2C6), anti-PARP (#9542), anti-phospho-c-Jun (D47G9), anti-phospho-JNK (81E11) and anti-NEMO (DA10-12), (Cell Signaling); anti-HOIL-1/C20ORF18, anti-HOIP/RNF31, anti-Tubulin (Sigma); anti-actin (sc-1616) (or Millipore), anti-iκBα (C15), IKK α/β (sc-7607), anti-NEMO (sc-8330), anti-p65 (sc372), anti-Ub (Santa Cruz Biotechnology) (P4D1), (Millipore (FK2) or Imgenex); anti-RIPK1 (BD Biosciences); anti-Sharpin, (ProteinTech); anti-HA, (Roche Diagnostics); anti-6His (Clontech) and anti-RGS4His (QIAGEN). The Met1-linkage specific Ub chain antibody was kindly provided by Vishva Dixit (Genentech). A Fam105B/OTULIN antibody was generated by Cambridge Research Biochemicals from rabbits that were immunized with full-length recombinant His6-OTULIN. Recombinant human TNFα (R&D systems); poly(I:C) and blasticidine (Invivogen;, zeocin (Invitrogen); G418 (PAA) and GTM20 GFP-Trap (Chromotek).

DNA oligos were obtained from Sigma Aldrich and siRNA duplexes against OTULIN and HOIP from Dharmacon (Smart pools, SP, Thermo Scientific), Sigma and Eurogentech.

The pRL-TK Renilla (Invitrogen) and the M3P sin rev κB firefly luciferase (coding for 4 NF-κB binding sites in the firefly luciferase promoter) plasmids were a kind gift of Felix Randow (MRC LMB), pcDNA3-HOIP1-/HOIL1-/Sharpin-V5-His vectors were kindly provided by Henning Walczak (Imperial College, London). pcDNA4/TO/N-MRGS6H (a modified pcDNA4/TO/myc-His B, Invitrogen) was a kind gift of Gerrit J.K. Praefcke (University of Cologne). pEBG-NEMO FL was a kind gift from Sally Swift (ICR London).

Molecular Biology

The coding sequence for full-length Fam105B/OTULIN was obtained following PCR amplification of a Human Adult Normal Brain cDNA library (Invitrogen) using KOD HotStart DNA polymerase (Novagen) and the following primers: OTULIN fwd 5'- AAGTTCTGTTT CAGGGCCCGATGAGTCGGGGGACTATGCCCC and OTULIN rev 5'-ATGGTCTAGAAAGCTTTATAGACTGGTCTCCTCACACA CTCTG. The PCR product was cloned into pOPINF and pOPIN-GFP (Berrow et al., 2007) using Infusion HD (Clontech). The OTULIN catalytic domain (OTULINcat, aa 80-352) was subcloned into pOPINF from full-length OTULIN using OTULIN80 fwd primer 5'-AAGTTCTGTTTCAGGGCCCGTTAAGCGTAGCTCCTGAAATGGATATCATGG-3'. OTULIN mutants (C129A, Y91F, W96A, L259E, E314R, and a triple mutant TQK100-102 to AAA) were generated in the full-length OTULIN sequence in pOPIN-F by site directed mutagenesis using the Quikchange method with KOD HotStart DNA polymerase according to manufacturer's protocol, and used for bacterial protein expression as well as for luciferase analysis. NEMO mutants were generated in pEBG-NEMO. OTULIN FL variants were subcloned into a modified pcDNA4/TO/MRGS6H using the following primers 5'-GCGGATCCATGAGTCGGGG GACTATGCCCC and 3-GGCTCGAGTTATCATAGACTGGTCTCCTCACACACTCTG. HA-tagged OTULIN (HA-OTULIN) was generated by PCR amplification of OTULIN from pcDNA4/TO/MRGS6H-OTULIN and cloning into Not1 and XhoI sites in pcDNA5/FRT/TO/N-2xHA-2xStrepTag (a kind gift from Pascal Meier and Tencho Tenev, Institute of Cancer Research, London, UK) using the following primers 5'-AAAAGCGGCCGCATGAGTCGGGGGACTATGCCC and 5'-AAACTCGAGTCATAGACTGGTCTCCTCACA CACTC. Ub and Met1-diUb was expressed from pET17 plasmids (Komander et al., 2009). Mutated (G76S) Met1-tetraUb was generated by cloning Ub fragments in which the C-terminal Gly-Gly coding sequence was exchanged for a BamHI site encoding Gly-Ser. FlAsH-tagged diUb constructs in which a C-terminal WCCPGCC motif replaces aa 72-76 were described previously (Ye et al., 2011). To generate untagged monoUb wild-type and E16A for mammalian expression, the following primers were used: pcDNA-Ub fwd GCGGATCCGCCGCCACCATGCAGATCTTCGTG AAGACCCTGAC and pcDNA-Ub rev CGAATTCTTATCACCCACCTCTGAGA CGGAGGAC. All constructs were verified by sequencing. Note that sequencing revealed that the pEBG-NEMO K285,309R construct had an additional E287G mutation.

Expression and Purification of OTULIN

OTULIN was expressed in *E. coli* strain Rosetta2 (DE3) pLacI. Cells were grown at 30°C in 2xTY containing 50 μg/ml ampicillin, 34 μg/ml chloramphenicol to an OD_{600} of 0.7. The culture was cooled to 20°C prior to induction with 150-400 μM IPTG and harvested 20 hr postinduction.

Cells were resuspended and lysed by sonication in lysis buffer (20 mM Tris pH 8.0, 500 mM NaCl, 50 mM imidazole, 5 mM β-mercaptoethanol, lysozyme DNaseI (Sigma), protease inhibitor cocktail (Roche)). OTULIN was purified by immobilized metal-affinity chromatography using Ni^{2+} resin (GE Life Sciences). The His6-tag was removed by overnight incubation with 3C Protease. For

biochemical studies, OTULIN was subjected to size exclusion chromatography (SEC) (HiLoad 16/60 Superdex 75, GE Life Sciences) in buffer A (20 mM Tris, 150 mM NaCl, 5 mM β-mercaptoethanol, pH 8.0). For crystallographic analysis, OTULINcat was dialyzed into 20 mM Tris, 5 mM DTT (pH 8.5) and subject to anion exchange chromatography (HiTrapQ FF, GE Life Sciences), prior to SEC in buffer A. Purified proteins were concentrated and flash frozen.

Qualitative DUB Linkage Specificity Assay

Qualitative deubiquitination assays were performed as previously described (Komander et al., 2009). Briefly, DUBs were diluted in 25 mM Tris (pH 7.5), 150 mM NaCl, and 10 mM DTT and activated at 23°C for 10 min. Subsequently, 1 μM di- or tetraUb was incubated with 10 nM, 50 nM or 1 μM DUBs in 50 mM Tris (pH 7.5), 50 mM NaCl and 5 mM DTT at 37°C. Samples were taken at different time points and directly mixed with 4x SDS sample buffer to stop the reaction. The reaction was resolved on 4%–12% SDS-PAGE gradient gels using MES running buffer (Invitrogen), and visualized by silver staining using the BioRad SilverStain Plus kit.

Ub Suicide Probe Generation and Analysis

Ub thioester was generated following Ub-intein cleavage as described previously (Akutsu et al., 2011). Haloalkylamine haloacid salts of 2-chloroethylamine (C2Cl), 2-bromoethylamine (C2Br) and 3-bromopropylamine (C3Br) were dissolved to a final concentration of 0.7 M in 20 mM Tris, 200 mM NaCl, 5 mM DTT, pH 8.0 and 0.2 mmol of each salt was mixed with 40 μM Ub-thioester. The reaction was initiated with 100 μl of 2 M NaOH and incubated at room temperature for 40 min. The reaction was quenched following addition of equimolar HCl and the reacted Ub-probes were buffer exchanged into 25 mM Tris, 200 mM NaCl, 5 mM DTT, pH 8.0.

For monitoring Ub-probe reactivity, vOTU was used as a positive control, which displays strong reactivity toward different Ub-probes (Akutsu et al., 2011). Enzymes were diluted to a concentration of 3.6 μM and reacted with 15 μM Ub-probe for 30 min at room temperature. The reaction was stopped by the addition of SDS sample buffer (Invitrogen) and resolved by SDS-PAGE.

Crystallization

Initial hits of all crystals were obtained by sitting-drop vapor diffusion method. Native (15 mg/ml), selenomethionine (SeMet)-substituted (0.1 mg/ml) and D336A (12 mg/ml) OTULINcat crystals were grown from drops containing an equal volume of protein and reservoir (100 mM MES/imidazole, 30 mM $MgCl_2$, 30 mM $CaCl_2$, 10% (w/v) PEG 4k, 20% (v/v) glycerol, pH 6.5). For SeMet-substituted crystals, native crystals were used to seed hanging drops containing SeMet-substituted OTULINcat. For Met1-diUb complex crystals, OTULINcat C129A was mixed with Met1-diUb in a 1:1.2 molar ratio, and set up at a concentration of 13 mg/ml. Crystals grew from 100 mM Bis-Tris, 2 M $(NH_4)_2SO_4$, pH 6.5. Prior to data collection WT, D336A and SeMet-OTULINcat crystals were harvested and vitrified in liquid nitrogen. OTULINcat C129A-Met1-diUb crystals were soaked in 2.5 M Li_2SO_4 prior to vitrification.

Data Collection, Structure Determination, and Refinement

Diffraction data were collected at the European Synchrotron Radiation Facility (ESRF), beam lines ID14-4 and ID23-1, and at Diamond Light Source, beam line I04-1. Diffraction images were processed and integrated using iMOSFLM (Battye et al., 2011) and scaled using SCALA (Evans, 2006). The structure of OTULINcat was determined by SAD phasing using data collected from a SeMet-substituted crystal. Phasing and density modification were performed using the Shelx Software pipeline (Sheldrick, 2008). Automated model building using ARP/wARP (Langer et al., 2008) fitted ~95% of the OTULIN sequence into the electron density. The structure of OTULINcat D336A was determined using a refined model of the apo OTULINcat structure with residues from the catalytic site omitted during the early stages of refinement. The structure of OTULINcat C129A in complex with Met1-diUb was determined by molecular replacement using PHASER (McCoy et al., 2007) with OTULINcat and Ub as initial search models. Iterative rounds of model building and refinement were performed with coot (Emsley et al., 2010) and PHENIX (Afonine et al., 2012), respectively. Final refinement of WT and D336A OTULINcat structures employed Refmac5 (Murshudov et al., 2011) where the occupancies of Cys129 and His339 were independently refined. Simulated-annealing composite omit maps were calculated using PHENIX (Afonine et al., 2012). All structural figures were generated with Pymol (www.pymol.org). Data collection and refinement statistics can be found in Table S1.

Expression, Purification, and Labeling of Ub Constructs

Ub constructs (mono- and Met1-diUb) were expressed and purified as described (Pickart and Raasi, 2005). In the case of FlAsH-tagged diUb constructs, 5 mM β-mercaptoethanol was included in all purification steps to prevent oxidation of the WCCPGCC motif. FlAsH-tagged Met1-diUb constructs were labeled by Lumio Green (Invitrogen) as described in (Ye et al., 2011), flash-frozen and stored in aliquots at −80°C.

Ub-AMC Assay

Ub-AMC (Boston Biochem) was diluted in reaction buffer (20 mM Tris, 100 mM NaCl, 1 mM β-mercaptoethanol, pH 7.4). For each reaction 10 μl of diluted substrate in a black 384-well low volume plate (Corning) was mixed with 10 μl of either: 10 pM UCH-L3 (Virdee et al., 2010) or 500 nM OTULIN at 37°C. The rate of AMC generation was measured using a PheraStar plate reader (BMG Labtech).

Fluorescent intensities were recorded following excitation at 340 nm and emission at 440 nm. Initial rates of Ub-AMC cleavage were recorded at each substrate concentration and fitted to a Michaelis-Menten equation using Graphpad Prism 5.

Binding Studies Performed with Size Exclusion Chromatography

Analytical SEC analysis was performed on an AKTA Micro system (GE Life Sciences) using a Superdex 75 PC 3.2/30 column equilibrated in SEC buffer (20 mM Tris, 50 mM NaCl, 5 mM DTT pH 8.0). Inactive OTULIN 80-352 C129A (OTULINcat C129A) was mixed with Met1-diUb variants in a 1:1.3 molar ratio (33.5 μM: 43 μM) and incubated at room temperature for 30 min. 125 μg of complex was loaded onto the column. Fractions containing protein were mixed with SDS loading buffer prior to SDS-PAGE analysis.

Binding Studies Performed with Fluorescence Anisotropy

To measure binding affinities of OTULIN C129A variants to Met1-diUb variants, 10 μl of 100 nM FlAsH-tagged Met1-diUb was aliquoted into a 384 -well low volume plate (Corning). Serial dilutions in FlAsH-buffer (20 mM Tris (pH 7.4), 100 mM NaCl, 5 mM β-mercaptoethanol, 0.1 mg/ml bovine serum albumin) were prepared of OTULIN C129A variants and 10 μl of this was added to FlAsH-tagged Met1-diUb containing wells. Fluorescence polarization was recorded on a PheraStar plate reader (BMG Labtech) using an optics module with λ_{ex} = 485 nm and λ_{em} = 520 nm. Fluorescence polarization values were fitted to a one-site binding model using Graphpad Prism 5 to derive binding constants (K_D).

Chain Cleavage Kinetics Assessed with Fluorescence Anisotropy

Change in fluorescence anisotropy upon cleavage of the peptide bond between the distal and proximal ubiquitin molecules was used to derive Michaelis-Menten rates (Virdee et al., 2010). Reactions were performed in a black 384-well low volume plate (Corning) and measured on a PheraStar plate reader (BMG Labtech) carrying a fluorescence polarization module with λ_{ex} = 485 nm and λ_{em} = 520 nm. Met1-diUb variants were serially diluted into FlAsH-buffer and contained a fixed concentration of 300 nM FlAsH-tagged Met1-diUb, to detect changes in fluorescence anisotropy. To each well, 10 μl of substrate was mixed with 10 μl OTULIN variants in FlAsH-buffer. The change of fluorescence anisotropy was recorded over a period of 5 min. The observed fluorescence polarization values were converted to percentage of substrate cleavage by comparing to baseline values from intact substrate and FlAsH-tagged monoUb that were acquired for each experiment. All measurements were corrected by subtracting changes in fluorescent anisotropy for FlAsH-tagged Met1-diUb alone. Triplicate recordings were made for each substrate concentration. Kinetic analysis was performed in Graphpad Prism 5. Initial rates of the enzymatic reaction were calculated and plotted against substrate concentration at a fixed OTULIN concentration, allowing determination of Michaelis-Menten parameters (Figure 3).

Linear Chain Assembly Assays

The RBR and C-terminal region of HOIP (residues 699–1,072) (Smit et al., 2012; Stieglitz et al., 2012) was cloned into pOPINK. Protein was expressed in *E. coli* Rosetta2 pLacI cells. Cells were grown at 37°C until an OD of 0.9, induced with 50 μM IPTG, 200 μM $ZnCl_2$ and grown at 19°C overnight. Cells were lysed in GST lysis buffer (270 mM sucrose, 10 mM glycerol 2-phosphate, 50 mM NaF, 14 mM β-mercaptoethanol, 50 mM Tris (pH 8.0)) and the lysate was incubated with Glutathione Sepharose 4B (GE Life Sciences). Bound protein was cleaved following overnight incubation PreScission protease. Cleaved protein was subjected to SEC (HiLoad Superdex75 16/60, GE Healthcare) in 200 mM NaCl, 10 mM DTT, 25 mM Tris pH 8.5.

The ligation assay was performed by mixing 100 nM E1, 2.2 μM UBE2L3, 2.5 μM HOIP 699-1072 and 0.25 mg/ml Ub or Ub E16A in reaction buffer (40 mM Tris pH 7.5, 10 mM $MgCl_2$, 0.6 mM DTT). A sample was taken before addition of 10 mM ATP (t = 0 min). The reaction was incubated at 37°C for 60 min before another sample was removed. Samples were analyzed by SDS-PAGE and stained using SilverStain Plus kit (BioRad).

Cell Culture

HEK 293ET, HeLa, U2OS, HCT116 and MCF7 cells were cultured in DMEM + GlutaMAX-I (GIBCO), RKO and Jurkat cells in RPMI (GIBCO) both supplemented with 10% (v/v) FBS (Hyclone, Thermo Scientific), 100 U/ml penicillin,100 μg/mL streptomycin at 37°C and 5% CO_2. T-REx293 cells (Invitrogen) were grown at similar conditions in DMEM + GlutaMAX-I (GIBCO), 10% (v/v) Tet-approved FBS (Clontech), 100 U/ml penicillin, 100 μg/ml streptomycin, 5 μg/mL blasticidin (InvivoGen).

Immunoprecipitation of Endogenous OTULIN from HEK 293ET Cells

Endogenous OTULIN was immunoprecipitated from HEK 293ET cells using OTULIN-specific antibodies coupled to Protein A sepharose (Roche) via Dimethylpimelimidate (Sigma). Cells were lysed in PBS, 0.1% (v/v) Triton X-100, COMPLETE protease inhibitor cocktail (Roche), 2 mM NEM and lysates were incubated overnight with Protein A-coupled OTULIN antibodies. Beads were washed five times with PBS, 0.01% (v/v) Triton X-100, COMPLETE protease inhibitor cocktail (Roche), 2 mM NEM and eluted in PBS/0.5% SDS pH 9.0 or by boiling in 2x SDS sample buffer.

Immunoprecipitation of Endogenous NEMO

HeLa cells were transfected and treated as indicated. Cells were lysed in IP buffer (25 mM HEPES pH 7.4, 150 mM KCl, 2 mM $MgCl_2$, 1 mM EGTA, 0.5% (v/v) Triton X-100) supplemented with 5 mM N-Ethylmaleimide (NEM; Sigma Aldrich), COMPLETE protease

inhibitor cocktail (Roche) and PhosSTOP (Roche) for 30 min on ice. Lysates were cleared by centrifugation and were incubated at 4°C overnight with anti-iKK γ-coupled beads (Santa Cruz Biotechnology). Beads were washed four times in 500 μl ice-cold IP buffer and bound material eluted with 0.2 M glycine, pH 2.5.

Purification of Ubiquitinated Proteins

Ubiquitin conjugates from cell lysates were pulled down in U2OS FlpIn-T-REx cells using affinity reagents. For isolation of Met1-Ub chains, recombinant protein containing one copy of the UBAN domain from human NEMO (residues 257-346) fused to Glutatione-S-transferase (GST) was used (M1-SUB). TUBE1, consisting of four UBA domains in tandem fused to GST, was used to purify all ubiquitin chains. One confluent 10 cm dish per condition was lysed in 600 μl Lysis buffer (20 mM Na_2HPO_4, 20 mM NaH_2PO_4, 1% (v/v) NP-40, 2 mM EDTA) supplemented with 1 mM DTT, 5 mM NEM, COMPLETE protease inhibitor cocktail and PhosSTOP. Lysates were divided in two and 50 μg TUBE1 or 100 μg M1-SUB was added. Lysates were cleared, mixed with Glutathione Sepharose 4B beads (GE Healthcare) and incubated at 4°C for a minimum of 2 hr with rotation. Beads were washed four times in 500 μl ice-cold PBS Tween-20 (0.1% (v/v)). Bound material was eluted with 1xSDS sample buffer.

Isolation of OTULIN-GFP from Cells for Specificity Assays

2×10^7 HEK 293ET cells were transiently transfected with pOPIN-GFP-OTULIN WT or C129A for 24 hr using GeneJuice (Novagen). Cells were lysed in 200 μl GFP lysis buffer (10 mM Tris pH 7.5, 150 mM NaCl, 0.5 mM EDTA, 0.5% (v/v) NP40, COMPLETE protease inhibitor cocktail (Roche)) for 30 min on ice. Diluted lysates were incubated with 120 μl equilibrated Magnetic GFP-Trap beads (Chromotek) for 16 hr at 4°C and washed following the manufacturer's protocol. Beads were then washed twice in DUB dilution buffer (25 mM Tris pH 7.5, 150 mM NaCl, and 10 mM DTT) and specificity assays were performed as described above using approximately 500 nM GFP-OTULIN bound to GFP-Trap beads.

Generation of Stable Inducible Cell Lines

T-REx293 cells were stably transfected with pcDNA/TO/N-MRGS6H-OTULIN using Gene Juice (Novagen). Selection was carried out with 150 μg/ml zeocin (InvivoGen) and individual colonies were sub-cloned, expanded and screened for OTULIN expression. OTULIN expression was induced with 1 μg/ml doxycycline for 24 hr.

For stable cell lines inducibly expressing nontargeting or OTULIN targeting miRNA, the respective sequences were cloned into the mammalian pT-REx-DEST30 plasmid following the BLOCK-IT Pol II miR RNAi Expression Vector Kit with EmGFP protocol (Invitrogen). The following sequences were used: OTULIN miRNA top strand 5′-TGCTGTGCTGTTGAATCCAGACCCAAGTTTTGGC CACTGACTGACTTGGGTCTATTCAACAGCA-3′, OTULIN miRNA bottom strand 5′-CCTGTGCTGTTGAATAGACCCAAGTCAGT CAGTGGCCAAAACTTGGGTCTGGATTCAACAGCAC-3′. A nontargeting control miRNA sequence was used according to the manufacturer's protocol. After stable transfection, selection was carried out with 500 μg/ml G418 (PAA) and individual clones were screened for GFP expression and OTULIN downregulation. OTULIN knock down was induced by 1 μg/ml doxycycline for 60-72 hr.

Transient Knockdown Studies

For transient knockdown studies, 20 nM siRNA duplexes against OTULIN or HOIP were transfected into HEK 293ET or U2OS cells using INTERFERin siRNA transfection reagent (Polyplus transfection) or Lipofectamine RNAiMAX (Invitrogen) according to the manufacturer's instructions and analyzed 48 hr posttransfection. For RNA interference in Jurkat T cells, 100 nM siRNA was transfected with Atufect transfection reagent (0.5–1 mg/ml) (Silence Therapeutics) and analyzed after 72 hr.

siRNA Sequences

Human Fam105B/OTULIN ON-TARGETplus SMARTpool (SP, Dharmacon/Thermo Scientific) contained the following siRNAs: GCUUAACUGUCUCGGGAAA, GGGCAUCAGAACCGAGAUU, UAGCAAAGGCAGGGCGCAA, CUUUAGUAGUAACGGGUUU. HOIP1/RNF31 ON-TARGETplus SMARTpool contained the following siRNAs: GCAGAAUACUCAUCCAAGA,CCUAGAACCUGAU CUUGCA, GGCGUGGUGUCAAGUUUAA, GCCGAGAUGUGCUGCGAUU. The ON-TARGETplus Nontargeting siRNA #1 (Dharmacon/Thermo Scientific) or sequence GGGAUACCUAGACGUUCUA served as a nontargeting control. Single siRNAs (SIGMA, Eurogentech) had the following sequences: #1: GACUGAAAUUUGAUGGGAA, #2: CAAAUGAGGCGGAGGAAUA, #3: ACAGAUAGCU UGUGAUGAA, #4: GCAUCAGAACCGAGAUUAA. Sequence #4 partly overlaps with one of the SP sequences.

NFκB Activity Assays

Cells were transiently transfected with the M3P sin rev κB firefly and pRL-TK Renilla (Promega) luciferase plasmids, and with plasmids encoding indicated proteins (pOPINF-OTULIN variants, pOPINF-A20, and/or LUBAC composed of pcDNA3.1-HOIL-1, pcDNA3.1-HOIP1 or pcDNA3.1-SHARPIN using Genejuice (Novagen). Cells were lysed in Passive Lysis Buffer (Promega) 15 or 24 hr posttransfection and luminescence was measured using a microplate reader (Berthold Detection Systems). For knockdown studies, cells were transfected with siRNAs (see above) 48 hr before lysis. After 24 hr, luciferase plasmids were transfected either alone or together with LUBAC; luciferase activity was assessed as described above. TNFα or poly(I:C) stimulation was carried out using the indicated concentrations for 6 hr or 20 hr, respectively.

Immunofluorescence Staining and Confocal Microscopy

HeLa cells were grown on Glass CultureSlides (BD Biosciences) and transfected with pcDNA4/TO/N-MRGS6H-OTULIN WT, C129A, W96A or L259E 24 hr prior to stimulation with 20 ng/ml TNFα for 30 min. Stimulation was stopped by adding ice-cold PBS. Cells were fixed in PBS, 4% paraformaldehyde (w/v), permeabilized in PBS, 0.3% (w/v) Saponin and blocked with PBS, 0.3% (w/v) Saponin, 4% (w/v) BSA. Primary and Alexa labeled secondary antibodies (Invitrogen) were applied in blocking buffer. Slides were embedded in Mounting Medium with DAPI (VECTOR laboratories) and data were obtained using a LSM 710 on the inverse Axio Observer AX10 microscope and the ZEN2009 software (Zeiss).

Isolation of GST-NEMO Variants from Stable Cell Lines

GST-NEMO WT or K285/309R (KR) was transfected with or without LUBAC into Doxycycline induced control cells or OTULIN WT overexpressing cells. 24 hr later, cells were lysed in PBS, 0.1% (v/v) Triton X-100, COMPLETE protease inhibitor cocktail (Roche), 2 mM NEM and 2.5 mg of total protein for each sample was incubated with 50 μl GSH-coupled sepharose beads 4B (GE Healthcare) overnight (o/n). Beads were washed five times with PBS, 0.01% Triton X-100 (v/v), COMPLETE protease inhibitor cocktail (Roche), 2 mM NEM and proteins were eluted by boiling in 2x SDS sample buffer. Samples and inputs were subjected to SDS-PAGE and western blotting, and detection was carried out using indicated antibodies.

NF-κB Target Gene Analysis

Stable cell lines downregulating or overexpressing OTULIN and their respective control cell lines were induced for expression with 1 μg/ml doxycycline for 72 hr (miRNA) or 24 hr (overexpression), respectively. Finally, cells were stimulated with 10 ng/ml TNFα for the indicated times. Signaling was stopped by adding ice-cold PBS. Cellular mRNA was isolated using the RNeasy Mini Kit, QIAshredder and the RNase-Free DNase Set (all QIAGEN). 1 μg mRNA was then reverse transcribed (QuantiTect Reverse Transcription Kit, QIAGEN) and cDNA quantification was measured by real-time PCR using the QuantiFast SYBR Green PCR Kit (QIAGEN) in a RotorGene 6000 (Corbett Research). Data were analyzed with the RotorGene 6000 software and visualized in Excel (Microsoft). All samples were normalized to their respective GAPDH levels.

Electrophoretic Mobility Shift Assays

For electrophoretic mobility shift assay (EMSA), cells were lysed in whole cell lysis buffer (20 mM HEPES pH 7.9, 350 mM NaCl, 20% (v/v) glycerol, 1 mM $MgCl_2$, 0.5 mM EDTA, 0.1 EGTA, 1% (v/v) Nonidet P-40, 0.5 M NaF, 1 M DTT, 1 M β-glycerophosphate, 200 mM Na vanadate, and 50x COMPLETE protease inhibitor (Roche)) according to standard protocols. Electrophoretic mobility shift assays (EMSA) were performed by using a ^{32}P-dATP–labeled, double-stranded NF-κB oligonucleotide probe (5′-CAGGGCTGGG GATTCCCCATCTCCACAGG-3′). The samples were separated on native polyacrylamide gels prior to autoradiography.

Cell Viability Assays

Stable inducible cell lines overexpressing or downregulating OTULIN and their respective control cell lines were induced for expression with 1 μg/ml doxycycline. After 24 hr (overexpression) or 72 hr (miRNA downregulation), on day 0, cells were stimulated or not with 50 ng/ml TNFα for 24 hr in doxycycline containing media. On day 1, for samples of day 2-4, media was exchanged to doxycycline containing media without TNFα. Thereby, detached cells were collected and re-plated into their respective wells. For all samples, attached and detached cells were collected after the indicated days and counted using a Vi-Cell XR cell viability analyzer (Beckman Coulter). Cells were also stained with crystal violet to visualize the cell counting results. The staining procedure included fixing in PBS, 4% (v/v) paraformaldehyde for 5 min and staining in 0.05% Crystal Violet (Sigma) for 30 min.

Analysis of Signaling Cascades

To analyze TNFα-induced NF-κB signaling, stable cell lines downregulating or overexpressing OTULIN and their respective control cell lines were induced for expression with 1 μg/ml doxycycline for 72 hr (miRNA) and 24 hr (overexpression), respectively. Finally, cells were stimulated with 10 (miRNA) or 100 ng/ml TNFα (overexpression) for the indicated times. Addition of ice-cold PBS stopped signaling. Cells were lysed in ice-cold lysis buffer (PBS, 0.1% Triton X-100 (v/v), protease inhibitor cocktail (Roche), 2 mM NEM, PhosphoSTOP (Roche)) for 30 min on ice. Lysate protein concentration was measured by Bradford assay, and equal protein amounts were subjected to SDS-PAGE and western blotting. Analysis was carried out with the indicated antibodies.

Immunoprecipitation of the TNF Receptor Signaling Complex

TNF-RSC was purified from the indicated cell lines after stimulation with 100 ng/ml Flag-TNFα ((Human TNFα, from Alexis) for the indicated times. Ice-cold PBS was added to the plate to stop stimulation. Following lysis, TNF-RSC was purified by incubation with Flag M2 agarose beads (Sigma). TNFR was purified from the unstimulated sample by adding 1 μg of TNFα during lysis. The purified TNF-RSC was analyzed by western blotting for linear ubiquitination, HOIP and TNFR1.

Kinase Assay

For Kinase assays, Jurkat T cells were lysed in 900 μl co-IP buffer (25 mM HEPES pH 7.5, 150 mM NaCl, 0.2% (v/v) NP-40, 10% (v/v) glycerol, 1 mM DTT, 10 mM sodium fluoride, 8 mM β-glycerophosphate, 300 μM sodium vanadate and protease inhibitor cocktail).

After immunoprecipitation with NEMO antibody (Santa Cruz, sc-8330), pellets were washed and incubated in kinase assay buffer (20 mM HEPES pH 7.5, 10 mM $MgCl_2$, 20 μM ATP, 20 mM β-glycerophosphate, 50 μM Na vanadate, 1 mM DTT) in the presence of GST-IκBα (1-53) for 25 min at 37°C. After boiling in loading buffer, the kinase reactions were separated on SDS-PAGE and analyzed by autoradiography.

SUPPLEMENTAL REFERENCES

Afonine, P.V., Grosse-Kunstleve, R.W., Echols, N., Headd, J.J., Moriarty, N.W., Mustyakimov, M., Terwilliger, T.C., Urzhumtsev, A., Zwart, P.H., and Adams, P.D. (2012). Towards automated crystallographic structure refinement with phenix.refine. Acta Crystallogr. D Biol. Crystallogr. *68*, 352–367.

Akutsu, M., Ye, Y., Virdee, S., Chin, J.W., and Komander, D. (2011). Molecular basis for ubiquitin and ISG15 cross-reactivity in viral ovarian tumor domains. Proc. Natl. Acad. Sci. USA *108*, 2228–2233.

Battye, T.G.G., Kontogiannis, L., Johnson, O., Powell, H.R., and Leslie, A.G.W. (2011). iMOSFLM: a new graphical interface for diffraction-image processing with MOSFLM. Acta Crystallogr. D Biol. Crystallogr. *67*, 271–281.

Berrow, N.S., Alderton, D., Sainsbury, S., Nettleship, J., Assenberg, R., Rahman, N., Stuart, D.I., and Owens, R.J. (2007). A versatile ligation-independent cloning method suitable for high-throughput expression screening applications. Nucleic Acids Res. *35*, e45.

Bremm, A., Freund, S.M.V., and Komander, D. (2010). Lys11-linked ubiquitin chains adopt compact conformations and are preferentially hydrolyzed by the deubiquitinase Cezanne. Nat. Struct. Mol. Biol. *17*, 939–947.

Emsley, P., Lohkamp, B., Scott, W.G., and Cowtan, K. (2010). Features and development of Coot. Acta Crystallogr. D Biol. Crystallogr. *66*, 486–501.

Evans, P. (2006). Scaling and assessment of data quality. Acta Crystallogr. D Biol. Crystallogr. *62*, 72–82.

Hospenthal, M.K., Freund, S.M.V., and Komander, D. (2013). Assembly, analysis and architecture of atypical ubiquitin chains. Nat. Struct. Mol. Biol. *20*, 555–565.

Katoh, K., Misawa, K., Kuma, K.-I., and Miyata, T. (2002). MAFFT: a novel method for rapid multiple sequence alignment based on fast Fourier transform. Nucleic Acids Res. *30*, 3059–3066.

Langer, G., Cohen, S.X., Lamzin, V.S., and Perrakis, A. (2008). Automated macromolecular model building for X-ray crystallography using ARP/wARP version 7. Nat. Protoc. *3*, 1171–1179.

McCoy, A.J., Grosse-Kunstleve, R.W., Adams, P.D., Winn, M.D., Storoni, L.C., and Read, R.J. (2007). Phaser crystallographic software. J. Appl. Cryst. *40*, 658–674.

Pickart, C.M., and Raasi, S. (2005). Controlled synthesis of polyubiquitin chains. Methods Enzymol. *399*, 21–36.

Sheldrick, G.M. (2008). A short history of SHELX. Acta Crystallogr. A *64*, 112–122.

Söding, J. (2005). Protein homology detection by HMM-HMM comparison. Bioinformatics *21*, 951–960.

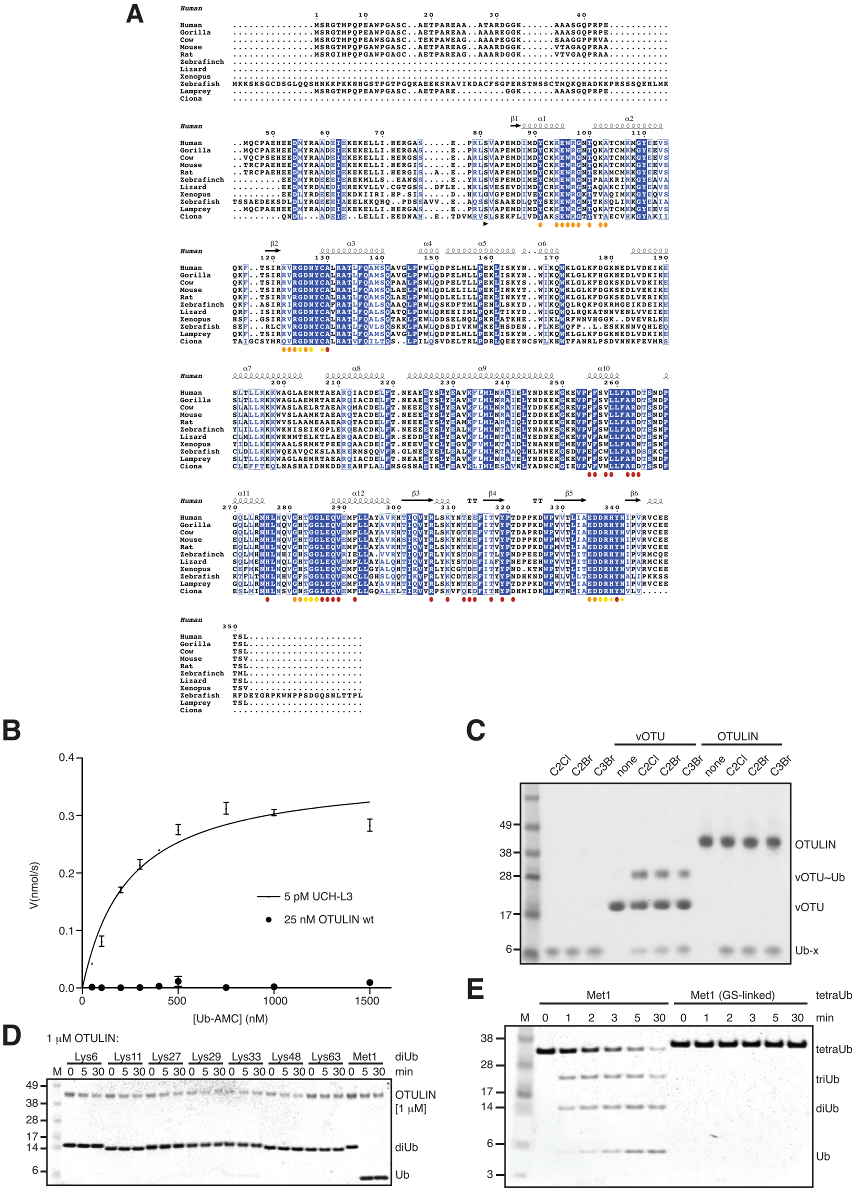

Figure S1. Multiple Sequence Alignment and In Vitro Analysis, Related to Figure 1

(A) Sequence alignment of FAM105B/OTULIN from various species, extracted from the Ensembl Genome Browser (http://www.ensembl.org/Homo_sapiens/Gene/Compara_Tree?g=ENSG00000154124;r=5:14664773-14699820). Several sequences (Zebrafinch, Lizard, *Xenopus*) lack an initiating N-terminal Met, suggesting that sequences were N-terminally truncated. Annotations of secondary structure are based on the Met1-diUb complex structure (Figure 2F). The annotation below indicates the start of the crystallized construct (black arrow), catalytic residues (yellow stars), and residues interacting with the proximal (orange circles), distal (red circles) or both Ub moieties (yellow circles) (see Figure 2G).

(B) Hydrolysis of Ub-AMC by 5 pM UCH-L3 and 25 nM OTULIN WT. Error bars represent the standard deviation from the mean calculated from triplicate measurements. See Extended Experimental Procedures for details.

(C) Ub-based suicide inhibitors. Ub-x, with x being a C2Cl, C2Br or C3Br warhead (Borodovsky et al., 2002), were prepared according to (Akutsu et al., 2011) and Extended Experimental Procedures and used to modify vOTU or OTULIN. A coomassie-stained SDS-PAGE gel is shown. While vOTU modification with Ub-probes results in a band shift due to the covalent modification, OTULIN is inert toward these reagents.

(D) Linkage specificity is retained with 1 μM OTULIN. DiUb (1 μM) of all possible linkage types is hydrolyzed over a time course by 1 μM OTULIN, and visualized on silver stained 4%–12% gradient SDS-PAGE gels.

(E) Incubation of OTULIN with Met1-linked tetraUb (Met1) or mutant Met1-linked tetraUb (Met1, GS-linked) in which Gly76 in the linkage is mutated to Ser. A silver stained SDS-PAGE gel of the indicated time course is shown. OTULIN hydrolysis of Met1-linked tetraUb immediately generates tri-, di- and monoUb, suggesting that OTULIN can cleave at any position within the tetraUb chain (endo-activity).

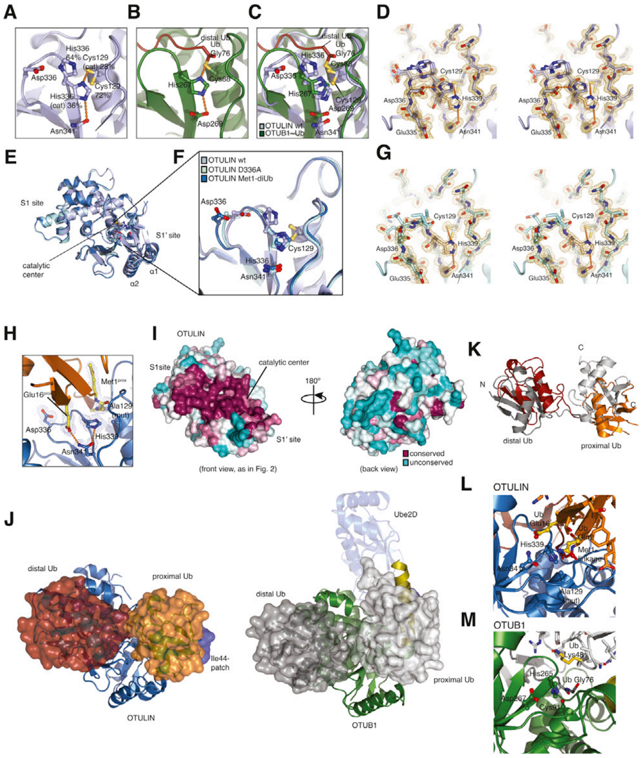

Figure S2. Structural Analysis of OTULIN, Related to Figure 2

The catalytic triad of OTULIN is delocalized in the absence of Met1-diUb.

(A) View of OTULIN catalytic triad as shown in Figure 2C, the occupancies of the catalytic cysteine (Cys129) and histidine (His339) are 28% and 36% respectively.

(B) View of the catalytic triad of OTUB1 (green) bound to Ub suicide probe (red) (PDB ID: 4DHZ) (Wiener et al., 2012).

(C) Superimposition of OTULIN and OTUB1 catalytic centers highlights the miss-orientation of OTULINs catalytic residues, His339 and Cys129 in the absence of Met1-diUb.

(D) Stereo view of the apo OTULIN catalytic site, as viewed from Figure 2C showing all residues within the region of the catalytic center enclosed in a 2|Fo|-|Fc| map, contoured at 1σ.

(E) Superimposition of OTULIN structures used within this study reveals no conformational changes between the OTULIN D336A and OTULIN WT structure (rmsd 0.24 Å) and OTULIN C129A from the Met1-diUb-containing structure and OTULIN WT (rmsd 0.76 Å).

(F) Close-up view of the various OTULIN catalytic centers. The catalytic His339 exists in two occupancies within the apo structure whereas only a single, catalytic conformation is observed in the D336A mutant structure. Likewise, within the OTULIN Met1-diUb structure His339 exists only in the catalytic conformation.

(G) Stereo view of the OTULIN D336A catalytic site enclosed in a 2|Fo|-|Fc| map, contoured at 1σ.

(H) Catalytic center of OTULIN Met1-diUb complex as shown in Figure 3E, with a 2|Fo|-|Fc| map contoured at 1σ shown for relevant residues.

(I) Surface conservation of OTULIN, based on the sequence alignment in Figure S1 generated with the Consurf server (http://consurf.tau.ac.il/). The surface of OTULIN (from the diUb complex structure, Figure 2F) is colored from green (no conservation) to dark red (fully conserved) and shown as in Figure 2G (left) as well as in a 180° rotation (right).

(J) Side-by-side comparison between the OTULIN and OTUB1 -diUb complexes, with OTULIN (blue, with red (distal) and orange (proximal) Met1-diUb) on the left and OTUB1 (green, with gray Ub molecules) on the right. The N-terminal helix in OTUB1 (yellow) binds to the Ile44 hydrophobic patch (blue on all Ub molecules) of the proximal Ub molecule. In the OTULIN structure, rotation of the proximal Ub leads to an exposed Ile44 patch of the proximal Ub moiety.

(K) Orientation of diUb molecules (colored as in (J) resulting from superposition of OTULIN and OTUB1. Although Ub molecules occupy similar spaces on the enzymes (see (J), both Ub molecules are rotated with respect to each other. Rotation of the proximal Ub is expected as different linkage points are involved (see position of C-termini, labeled C). The rotation of the distal Ub of ~18° is more surprising, and reveals that the S1 binding site of the enzyme also shows significant plasticity.

(L) Close-up of the OTULIN catalytic center bound to Met1-diUb. Glu16 and Gln2 of the proximal Ub are shown in yellow, and active site residues are labeled.

(M) Catalytic center of OTUB1 bound to two ubiquitin molecules. Lys48 of the proximal Ub is indicated, and is the only residue in close proximity to the catalytic center, explaining OTUB1's specificity. However, further Ub residues interacting with the catalytic triad as observed in OTULIN are not present.

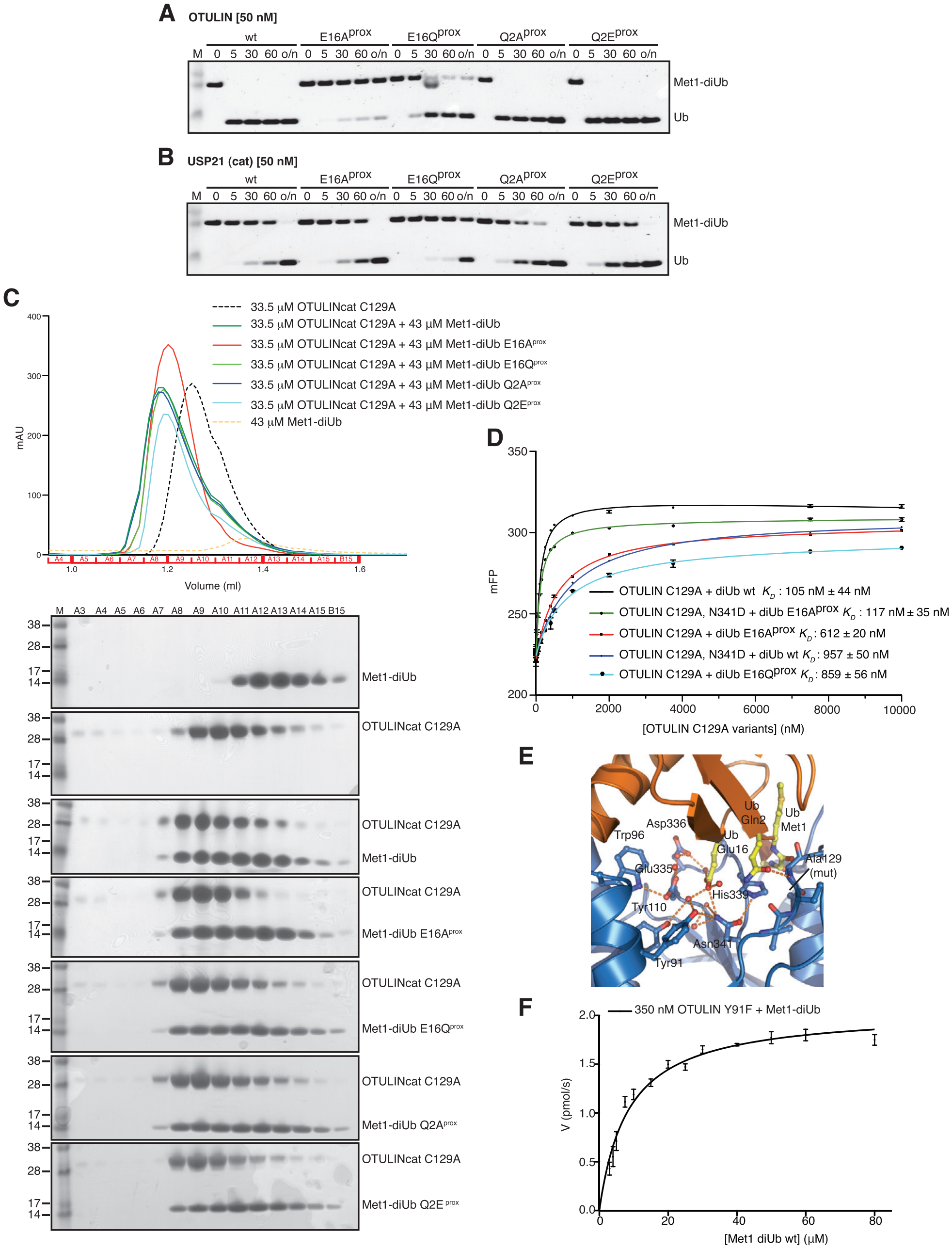

Figure S3. Mutant diUb Controls and Fluorescence Anisotropy Binding and Kinetic Studies, Related to Figure 3

(A) Met1-diUb and variants with point mutations in the proximal Ub moiety were hydrolyzed by OTULIN at 50 nM concentration (compared to 10 nM in Figure 3G). A time course of the reaction is resolved on a silver stained SDS-PAGE gel as in Figure 3G.

(B) Same as (A), but using the Ub specific protease USP21 (Ye et al., 2011) at 50 nM concentration.

(C) Analytical size exclusion chromatography (SEC) profiles of inactive OTULINcat C129A with Met1-diUb, and Met1-diUb variants containing point mutations in the proximal moiety. Experiments were performed with 33.5 μM OTULIN and 43 μM Met1-diUb mutants, giving a 1:1.3 molar ratio of OTULIN to diUb, respectively. Shifting of the SEC peak to the left indicates complex formation in all cases. Corresponding SDS-PAGE gels showing proteins in peak fractions according to the profile are shown below.

(D) Binding curves derived from fluorescence anisotropy measurements for OTULIN C129A and OTULIN C129A N314D using FlAsH-tagged Met1-diUb WT, E16A^{prox} and E16Q^{prox}. Error bars represent standard deviation from the mean of measurements performed in triplicate.

(E) View of the catalytic center of OTULINcat C129A bound to Met1-diUb, showing the interaction of Tyr91 with the catalytic Asn341. (F) Kinetic parameters determined from hydrolysis of FlAsH-tagged Met1-diUb WT by 350 nM OTULIN Y91F used to derive the rate constants shown in Figure 3K. Error bars represent standard deviation from the mean from experiments performed in triplicate.

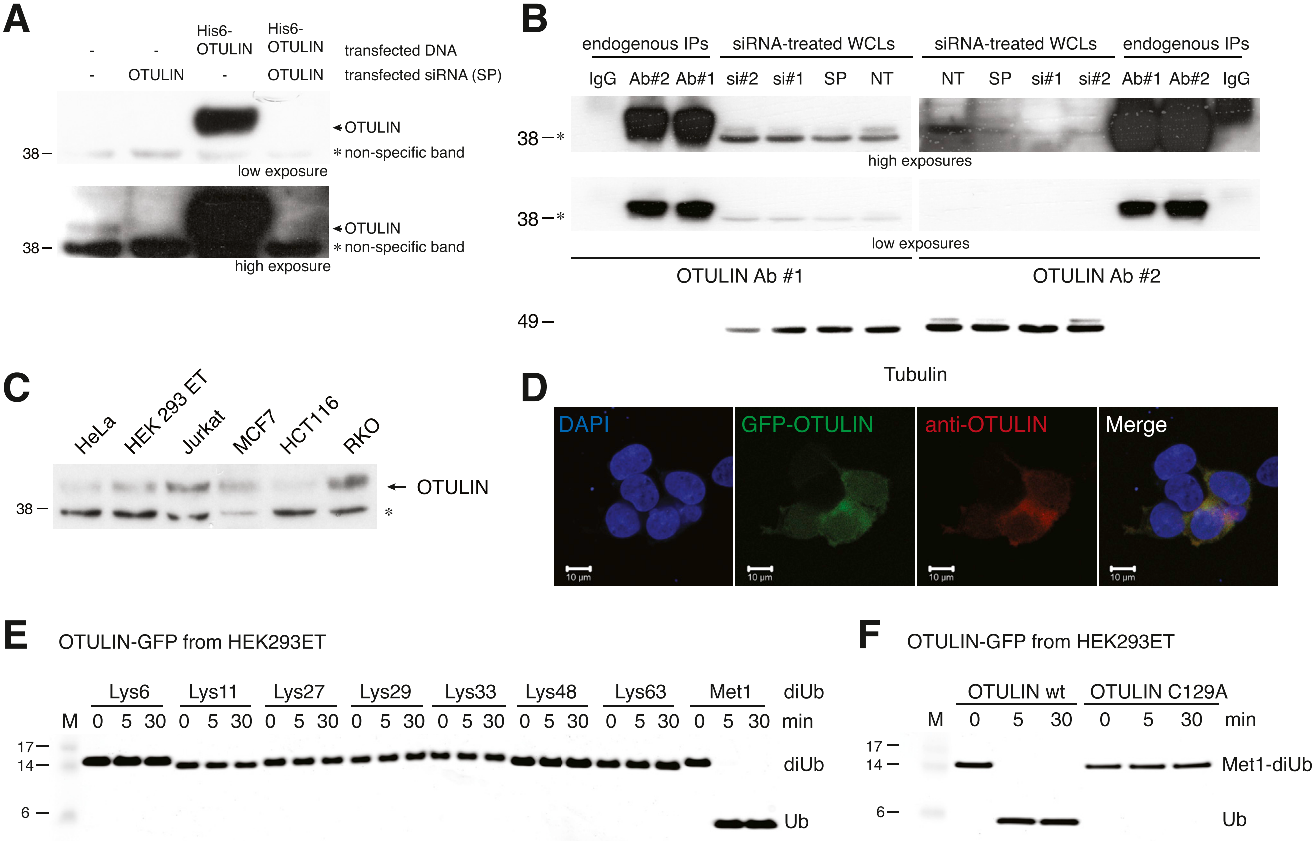

Figure S4. Reagent Validation, Cellular Localization of OTULIN, and Stable Inducible OTULIN Cell Lines, Related to Figure 4

(A) A polyclonal rabbit, anti-OTULIN antibody was generated that detects endogenous and overexpressed OTULIN in HEK 293ET cell lysates. siRNAs against OTULIN (Dharmacon pool) reduces OTULIN protein levels. Two exposures of the same western blot are shown.

(B) Comparison of the two polyclonal antisera obtained from rabbit immunization. Both antibodies detect an endogenous band that is reduced in siRNA-treated samples and that is also immunoprecipitated by both antibodies from HEK 293ET cells.

(C) OTULIN can be detected in all analyzed cell lines.

(A–C) *, nonspecific band.

(D) Localization of C-terminally GFP-tagged OTULIN in HEK 293ET cells. *Left*, DAPI stain of cell nuclei; *second from left*, GFP fluorescence; *second from right*, immunodetection of transfected OTULIN with anti-OTULIN antibody; *right*, merged image. Scale bar, 10 μm.

(E) GFP-tagged OTULIN was expressed in HEK 293ET cells and purified using GFP-Trap resin. Specificity assays were performed as in Figure 1D using approximately 500 nM GFP-OTULIN bound to GFP-Trap beads. A silver-stained 4%–12% SDS-PAGE gel is shown.

(F) GFP-tagged OTULIN C129A is catalytically inactive when expressed in HEK 293ET cells and purified by GFP-Trap resin.

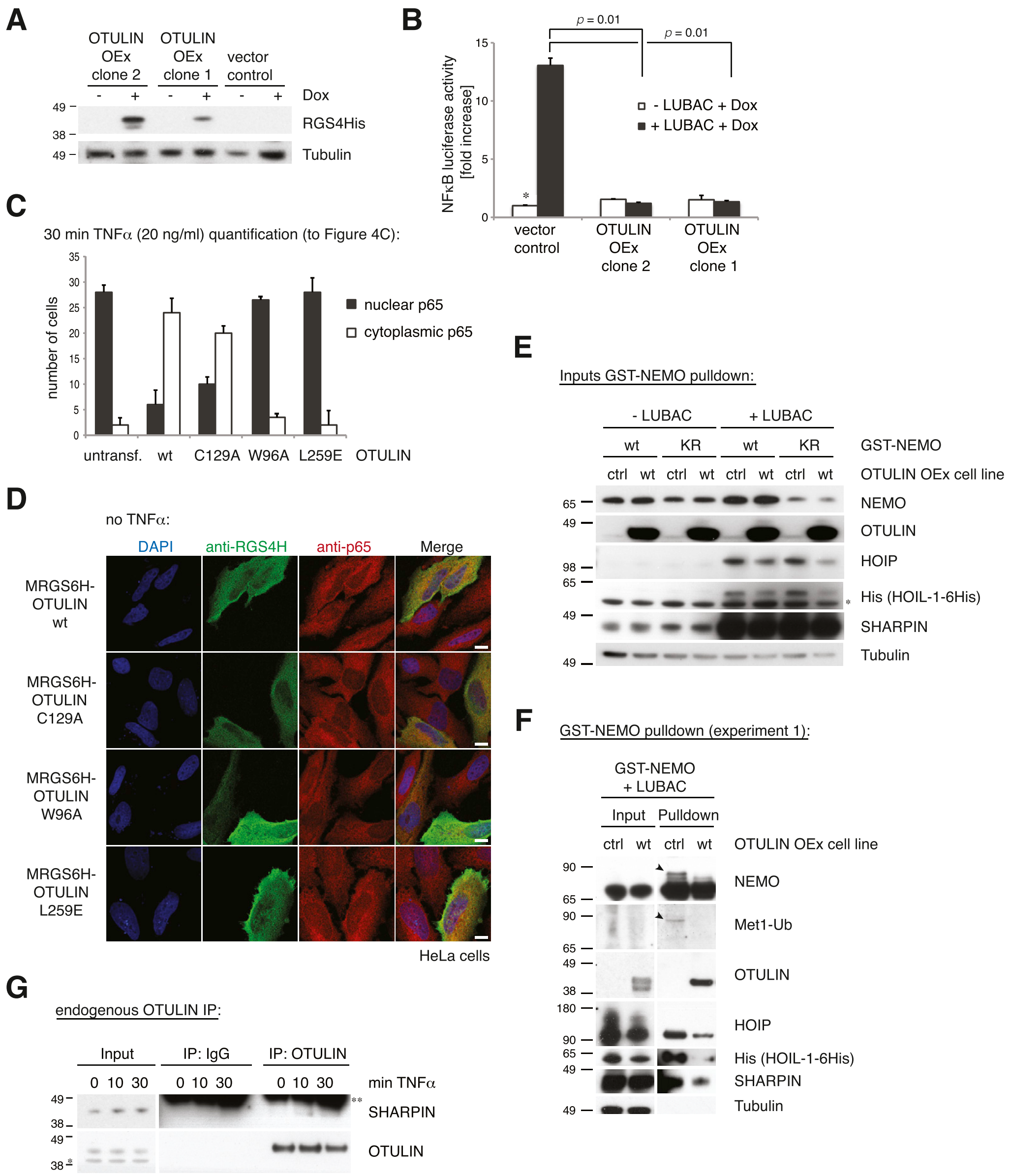

Figure S5. Stable Inducible OTULIN Cell Lines and Controls, Related to Figure 4

(A) Western-blotting analysis of T-REx293 cells stably expressing a doxycycline-inducible MRGS6His-tagged OTULIN construct.

(B) NF-κB luciferase activity of OTULIN-overexpressing cells in response to LUBAC coexpression. Error bars represent standard deviation of experiments performed in duplicate. p values are given to indicate significance and asterisks indicate mean values set to 1.

(C) Quantification for the experiment shown in Figure 4C.Black bars represent the number of HeLa cells in which p65 translocated into the nucleus while white bars represent the number of cells in which p65 was excluded from the nucleus after 30 min of TNFα stimulation (20 ng/ml) in cells transfected with the indicated OTULIN variants. n = 30 for each condition. Error bars represent standard deviation from the mean from duplicate measurements.

(D) Control to Figure 4C. HeLa cells were transiently transfected with indicated plasmids and analyzed in immunofluorescence with indicated antibodies without any prior TNFα stimulation (see Extended Experimental Procedures). Scale bar = 10 μm.

(E) Input levels of transfected proteins for pulldown of GST-tagged NEMO variants of Figure 4D in control or OTULIN overexpressing T-REx293 cell lines (see Extended Experimental Procedures) after western blotting with the indicated antibodies.

(F) Pulldown of GST-tagged wild-type NEMO after coexpression with LUBAC, in control or OTULIN overexpressing T-REx293 cell lines. Western blotting with indicated antibodies reveals polyUb on NEMO (arrowhead), which is lost when OTULIN is coexpressed. This was the first experiment showing that NEMO is modified by a Met1-linked polyubiquitin chain of a distinct length and is shown to support the results of Figure 4D.

(G) Immunoprecipitation of endogenous OTULIN (see Extended Experimental Procedures) coprecipitates endogenous SHARPIN before and after TNFα stimulation (100 ng/ml) as revealed by western blotting with the indicated antibodies.*, nonspecific band; **, heavy chain.

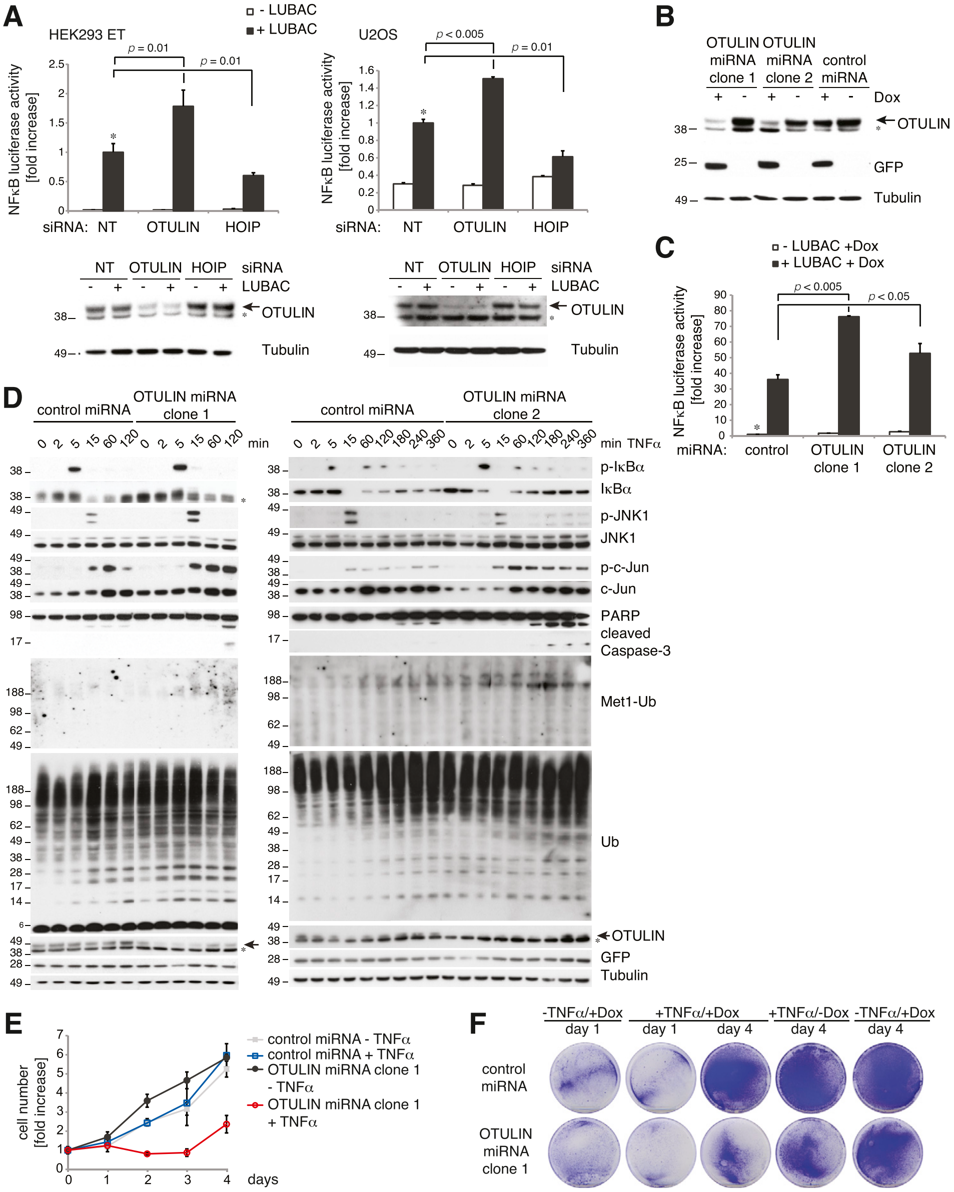

Figure S6. OTULIN Knockdown Studies, Related to Figure 7

(A) HEK 293ET (left) and U2OS (right) cell lines were transfected with NF-κB reporter plasmids, LUBAC and with siRNAs targeting OTULIN, HOIP or a nontargeting control siRNA. NF-κB reporter activity was measured as in Figure 4B. Error bars represent standard deviation from the mean of experiments performed in triplicate. p values are given to indicate significance and the asterisk indicate the mean value set to 1. Western blots with indicated antibodies show knockdown efficiency. Here, the asterisk indicates nonspecific bands.

(B) T-REx293 cells stably transfected with an inducible nontargeting control miRNA or with an inducible OTULIN-targeting miRNA are analyzed by western blotting with indicated antibodies. Doxycycline induces the OTULIN miRNA (together with GFP expression) from the GFP mRNA 3′UTR, and expression can be monitored by expressed GFP levels following doxycycline induction.

(C) LUBAC-induced NF-κB luciferase activity is increased in OTULIN-depleted cell lines. Error bars represent the standard deviation from the mean of experiments performed in duplicate. p values are given to indicate significance.

(D) Control or OTULIN-depleted cell lines were stimulated with TNFα (10 ng/ml) for the indicated times, and lysates were analyzed by western blotting with the indicated antibodies. *, nonspecific band.

(E) Cell viability counts of stable doxycycline-inducible control and OTULIN-miRNA expressing T-REx293 cells after treatment with and without doxycycline (+/− Dox, 1 μg/ml for 72 h) prior to TNFα treatment (+/− TNFα, 50 ng/ml for 24 hr) (see Extended Experimental Procedures). Error bars represent the standard deviation from the mean for experiments performed in triplicate.

(F) Clonogenic survival of cells treated as in (E).
